# Quantum-SpinalNet: a hybrid deep learning approach for mammographic breast cancer detection

**DOI:** 10.3389/fdgth.2026.1753820

**Published:** 2026-04-13

**Authors:** Martina Jaincy D E, Venkatasubbu Pattabiraman

**Affiliations:** School of Computer Science and Engineering, Vellore Institute of Technology, Chennai, Tamil Nadu, India

**Keywords:** breast cancer detection, hybrid deep learning, mammography, quantum neural network, Swin ResUnet3+

## Abstract

**Introduction:**

Breast cancer diagnosis in mammograms remains challenging due to limitations in preprocessing, accurate differentiation of benign and malignant cases, and precise tumor segmentation.

**Methods:**

We propose Quantum-SpinalNet, a hybrid deep learning model combining Swin ResUNet3+ for tumor segmentation with a Deep Quantum Neural Network (DQNN) and SpinalNet for classification. Preprocessing involves CEAMF-based denoising, Z-score normalization, and context-aware contrast enhancement using spatial energy curves. Swin ResUNet3+ integrates ResUnet3+ decoders with Swin Transformer encoders for effective tumor localization and context extraction.

**Results:**

Evaluation on the CBIS-DDSM and DDSM datasets demonstrates superior performance: accuracy 93.8%, sensitivity 94.1%, specificity 92.7%, precision 91.2%, F1 score 92.6%, Dice coefficient 0.89, and IoU 0.82.

**Discussion:**

The proposed Quantum-SpinalNet provides a robust and interpretable framework for mammographic breast cancer detection, improving segmentation and classification precision, and supporting clinical diagnostic workflows.

## 1 Introduction

Breast cancer remains the most frequent and deadliest cancer among women globally. According to the World Health Organization (WHO), breast cancer is the most commonly diagnosed malignancy in women and is responsible for the highest number of cancer-related fatalities worldwide. Early identification and accurate analysis are important to enhancing survival, as prompt treatment greatly increases the possibility of survival. In 2022 alone, approximately 2.3 million women were diagnosed with breast cancer, resulting in an estimated 665,684 deaths worldwide (1–5). Genetic predisposition plays a crucial role in the onset of breast cancer, often serving as a primary factor preceding other risk contributors such as obesity and certain medications. A variety of factors are associated with an increased risk of breast cancer, including hereditary and genetic influences, reproductive history, hormonal exposure (both endogenous and exogenous), lifestyle choices, anthropometric characteristics, increased breast density observable in mammographic imaging, and the presence of benign breast conditions. Comorbid conditions like heart failure (HF), ischemic stroke (IS), and coronary heart disease (CHD) may also increase the risk of breast cancer.

In addition to the physical health problems caused both by the disease and its treatment, there is also the financial and practical impact of breast cancer and a very meaningful negative impact upon mental health, emotional well-being, and quality of life (6–9). The early diagnosis of breast cancer is important in order to achieve a positive prognosis because breast cancers have a high mortality rate when diagnosed late and multiple screening and diagnostic tests have been developed and used for breast cancer detection and diagnosis (10–16). These include clinical breast exams, thermography, biopsy (tissue sampling), breast ultrasound imaging (BUSI), mammography, and breast magnetic resonance imaging (BMRI). To enable rapid and precise diagnosis, each technique has a distinct function in recognizing and describing breast abnormalities.

Although mammography utilizes a lower radiation dose compared to other diagnostic imaging techniques (17–20), it remains the most effective tool for the early detection of breast cancer. Mammograms are vital in catching breast tissue adjustments before signs and symptoms begin to increase. Via mammograms, radiologists are able to observe tiny abnormalities in breast structures (e.g., microcalcifications, breast lumps, or architectural distortions that may be a sign of a malignant or a benign tumor). However, mammograms are susceptible to human error, as the radiologist receives a visual record and must process this information themselves.

The advance of technology has seen the use of models such as image processing, machine learning (ML), and deep learning (DL) in medical imaging, thereby revolutionizing radiology, as they provide radiologists with a number of reliable tools for detecting breast cancer. These tools can detect patterns from mammogram images and diagnose any malignancies themselves, which means the tools can diagnose patients and reduce the load on, and potential mistakes made by, physicians. The benefit of integrating artificial intelligence (AI) and mammography is to lessen the frequency of mistakes and enhance the consistency of the reading procedure. As early as 1980, CAD systems (21–27) were used to augment human performance via the use of machines. They depended on guide layout functions and rule-based algorithms to diagnose suspicious regions. However, there is still some work to go to achieve a reliable accuracy rate.

The advent of machine learning (ML) and deep learning (DL) models (28–31), particularly convolutional neural networks (CNNs), has revolutionized the transition from manual feature extraction to automated feature identification. These networks could be utilized in image classification and segmentation as can process statistics and quickly identify any issues.

The advance of ML and DL models (28–31), specifically CNNs, has transformed the approach from manual feature extraction to automated feature learning. CNNs are specifically useful for image classification and segmentation as they can examine hierarchical representations of photograph information immediately from pixel records. For mammography, deep learning tools may be able to establish a clear difference between benign and malignant tumors. They can also localize the regions of best interest themselves and compare the volume of the tumor. Research has confirmed that DL-system models, when trained on large amounts of useful information, are able to attain a level of accuracy that is near or, in some cases, higher than that of especially skilled radiologists. However, the employment of AI-assisted breast cancer detection systems is facing numerous obstacles. A widespread issue is the supply of large, annotated mammograms for training, which continues to be inadequate. The process of annotating scientific images requires an expert, and this step is difficult and costly. Furthermore, exposure to specific imaging methods, equipment, and populace profiles can reduce the reliability of the algorithms trained. It is essential that these tools are trained on reliable data so that healthcare facilities can depend on the AI results.

While TransUNet and Swin-UNETR, two traditional hybrid models for breast cancer detection, are good at capturing global information through attention processes, they also have substantial computational costs and poor border segmentation. In contrast, the proposed Q-SpinalNet utilizes Swin ResUnet3+, which integrates residual connections, transformer-based attention, and a U-Net-style encoder-decoder architecture. Such an approach results in contextual global knowledge and detailed local features that can identify tumor borders accurately and cost-effectively. The Quantum Neural Networks (QNNs)-based classifiers, however, due to their quantum nature, suffer from the standard “black-box” behavior with very little interpretability. These models may perform better but are not currently clinically useful because they cannot be generally understood by physicians. Q-SpinalNet incorporates SpinalNet, an anatomically based model of human spinal cord processing. The modularity of the SpinalNet structure allows for analysis of the decision-making of individual layers. Lastly, the ability of conventional CNNs with attention processes to generalize across various tumor sizes and shapes is limited by their fixed receptive area and potential vulnerability to data imbalance. This is addressed by Q-SpinalNet, which enhances generalization and robustness performance by combining probabilistic learning from DQNN with context-aware feature fusion from SpinalNet to enable dynamic adaptation to heterogeneous data.

A hybrid Q-SpinalNet model that integrates quantum and spinal-layer processing was created and a novel three-stage preprocessing technique specifically designed for mammography data was suggested. Swin ResUnet3+ was introduced for accurate and contextually aware tumor segmentation; it demonstrated outstanding interpretability using attention maps and a biologically inspired logic flow. Its performance was verified on two datasets using statistical significance and ablation analysis.

### 1.1 Motivation

A primary focus of current research is the early and reliable detection of breast cancer through various imaging modalities (32–36). However, classification, segmentation, and cancer severity prediction continue to be major. obstacles for many current methods. The following are the main problems with the current body of work:

- A lack of emphasis on strong feature engineering, especially in selecting the most relevant features. This frequently results in redundant features, which increase computational load and reduce overall system performance.
- Most segmentation techniques rely on simple threshold-based methods, which are out of date and frequently produce a high number of false positives. While some research has used deep learning for more intelligent segmentation, these still have trouble defining tumor boundaries precisely and frequently overlook crucial areas like the pectoral muscle. Consequently, the result is a wide range of false alarms and increased ineffectiveness due to low true positive rates.
- While many strategies use basic preprocessing techniques like noise reduction or assessment improvement, only a small number of them use a comprehensive, step-by-step preprocessing pipeline. Preprocessing that eliminates noise and artifacts, modifies contrast, and lowers false positives in benign circumstances has only been addressed in a small number of investigations.

These difficulties draw attention to the need for more precise and automated methods of mammography-based breast cancer detection. With the current analysis, we want to improve detection accuracy by optimizing real positive costs, minimizing phony high-quality quotes, and utilizing state-of-the-art methods for image processing and evaluation. The CNN + Transformer topologies (TransUNet, Swin-UNETR, etc.) used in most current hybrid models are good at extracting features but struggle with computational complexity and precisely identifying tumor boundaries. Quantum Neural Networks (QNNs), which are often freestanding, are often not integrated with SpinalNet and other biologically inspired components. Our method is unique since it was the first to combine a quantum-inspired DQNN with a physiologically driven SpinalNet. That uses Swin ResUnet3+ to provide accurate tumor segmentation by combining residual connections and self-attention techniques. It incorporates physiologically realistic thinking to make judgments more clinically interpretable and robust in real-world diagnostics. Compared to traditional noise reduction or histogram-based methods, triple-stage preprocessing offers a more comprehensive workflow.

### 1.2 Motivation for introducing quantum-inspired learning

Traditional deep neural networks, such as CNN-based architectures and conventional SpinalNet, use deterministic real-valued transformations to model feature interactions in a strictly additive and hierarchical manner. Despite their effectiveness, these models frequently fail to capture non-linear decision boundaries and high-order feature correlations found in complicated medical imaging data, especially when training samples are few and class distributions overlap.

Richer representational capability is naturally supported by mathematical structures introduced by quantum physics, such as state superposition, complex-valued probability amplitudes, and interference effects. Therefore, improved feature interaction modeling inside a conventional computing environment is the driving force behind integrating a quantum-inspired framework rather than hardware acceleration.

### 1.3 What makes up quantum-SpinalNet's “quantum” component?

A physical quantum circuit is not simulated by Quantum-SpinalNet. Rather, it uses a neuronal representation influenced by quantum mechanics, where

- Complex-valued amplitudes are used to encode feature vectors as quantum-like states.
- Superposed feature states are represented by intermediate neuron activations, allowing for the simultaneous assessment of many feature interactions.
- Constructive and destructive interference, which suppresses redundant or noisy feature patterns while enhancing discriminative ones, affects the classification choice.

Because the decision boundary is formed by phase-dependent interactions rather than solely magnitude-based responses, this quantum-inspired formulation differs fundamentally from conventional activation functions.

### 1.4 Structural innovation: SpinalNet integration

The incremental, segment-wise design of SpinalNet is uniquely integrated with this quantum-inspired learning process in the proposed model:

1. **Incremental Feature Injection:** Unlike fully linked quantum-inspired networks, Quantum-SpinalNet applies quantum-inspired transformations locally at each spinal segment by processing data progressively.
2. **Decreased Parameter Complexity:** Compared to deep quantum-inspired multilayer networks, the spinal structure mitigates overfitting by limiting parameter increase.
3. **Progressive Quantum Feature Refinement:** To enable hierarchical interference-based feature augmentation, each spinal segment refines the quantum-encoded representation before forwarding it.

To the best of our knowledge, this is the first system for medical image classification that explicitly integrates interference modelling and quantum-inspired state encoding into a SpinalNet architecture.

### 1.5 Why classification performance is improved by quantum-SpinalNet

Three complementary variables contribute to performance improvement:

- **Improved feature separability:** Class-discriminative patterns that could be weak or overlapped in classical feature space are amplified by quantum interference.
- **Robustness to noise:** By distributing feature relevance over several associated dimensions, superposition lessens the model's susceptibility to noise and artifacts unique to a given dataset.
- **Better generalization with fewer parameters:** Coupling spinal architecture with quantum-inspired encoding enhances expressiveness without increasing network depth or parameter count.

As shown in the experimental results section, these benefits empirically translate into steady gains in accuracy, sensitivity, and specificity over traditional SpinalNet, CNN-based classifiers, and other non-quantum-based methods.

### 1.6 Differentiation from current classical and quantum-inspired models

Quantum-SpinalNet presents localized quantum-inspired processing inside an incremental learning framework, in contrast to previous quantum-inspired neural networks that concentrate on standalone quantum layers or global transformations. The advantages of quantum-inspired feature modeling are maintained while improved interpretability and computing efficiency are made possible by this structural differentiation.

### 1.7 Contribution

- **Novel Hybrid Classifier:** Q-SpinalNet is the first instance of a hybrid neural network combining multilayer biological networks with concepts of quantum computation.
- **Triple-Stage Preprocessing:** Unlike medical imaging denoising, CEAMF-based denoising is specific to mammograms.
- **Segmented Attention Integration:** Swin ResUnet3+ can adaptively retain global anatomical background and fine-grained detail simultaneously.
- **Clinical Interpretability:** Eased by a biological decision pathway simulation, attention maps, and confidence scores.
- **Better Performance Indicators:** Each component was comprehensively tested (*p*-value < 0.01 against baselines), with solid metrics and statistics.

## 2 Literature survey

An overview of the recent work on breast cancer detection using mammography is presented in Table 2, along with the techniques used, datasets employed, and the shortcomings of existing studies.

BreastNet18 is a deep learning model proposed by Sidratul et al that detects breast cancer at an early stage in mammogram images (37). Due to various preprocessing techniques and complete facts augmentation, the fine-tuned VGG16 model has achieved a 98.02% class accuracy on breast lesions. Furthermore, it is validated by ablation analysis and k-fold cross validation and can serve as reliable choice-support software for radiologists in the early diagnosis and treatment planning of breast cancer.

A dual directional CNN model that predicts breast cancer biomarker status based on mammographic images was proposed by Petrini et al. (38). It was trained using a combination of the digitized Database for Screening Mammography (DDSM) and the Curated Breast Imaging Subset of the DDSM, predicting tumor classification between benign and malignant. It was mostly built on EfficientNet-B4 with twin-view classifiers.

Soulami et al. (39) delivered a one-stage detection framework that utilized a U-Net-primarily based on digital mammogram images. This procedure resized mammograms and converted them to RGB layout to improve segmentation outcomes. The U-Net architecture classified images into normal, benign, and malignant categories for training, and its performance was validated using datasets such as DDSM and INbreast.Even as this technique showed utility in segmentation and detection, its accuracy was hindered by noise, image artifacts, and background muddle.

Rehman et al. (40) evolved an automatic CAD system that leveraged deep learning for breast cancer staging. Their technique featured pixel-wise segmentation to enhance detection accuracy but omitted pectoral muscle areas, which resulted in the loss of vital anatomical data. A depth-wise CNN was used to identify architectural distortions, however its recognition capability on segmented areas limited its potential to successfully extracting architectural capabilities, thereby reducing its detection functionality.

Ibrokhimov et al. (41) carried out a twin-path deep learning approach for classifying breast cancers in mammographic images. They improved their set of rules with datasets from the Breast Imaging-Reporting and Data System (BI-RADS) and the INbreast database. The process started by isolating breast regions and segmented tumor-like areas through a region of interest (ROI) extraction approach. These segmented patches were processed with a region-based convolutional neural network (R-CNN) for field detection. Duplicates were eliminated at this stage, and specific patches were delivered to the CNN classifier, which classified them into two classes, namely ordinary and malignant, based on severity.

Fahrozi et al. (42) utilized a CNN for breast cancer detection using mammographic images. A selected breast cancer dataset was employed to train the model. The images underwent preprocessing, together with resizing, and were then divided into training and testing sets. The preprocessed images were fed into the CNN, which classified them into three categories: malignant, benign, or ordinary.

Zahoor et al. (43) investigated the integration of optimization strategies with deep learning techniques for classifying breast cancers in mammograms. Their studies utilized three publicly available datasets: MIAS, INbreast, and CBIS-DDSM. The images were augmented to enhance data variability and volume. Features were extracted from those augmented images using two deep mastering techniques, namely NasNet mobile and MobileNet V2, resulting in useful function vectors. An improved version of the Whale Optimization set of rules was adapted to select the most applicable functions. These optimized functions were used to improve the evaluation of the severity of the diagnosed cancer.

The study also identifies numerous current research gaps in present day breast cancer detection strategies. Constraints in image quality, segmentation accuracy, and feature extraction are the main issues. The proposed technique primarily aims to address these issues by improving the clarity of mammographic images and improving both the segmentation technique and overall category efficacy. DQNN's strengths include capturing uncertainty through quantum probabilistic behavior, facilitating superposition-based feature representation and parallel processing, and recognizing abstract patterns, though it may not have biological plausibility or chronological ordering. SpinalNet's contribution is that it replicates the neuronal integration of the spinal cord by processing local features (from segmented images) over time. Together, they achieve a compromise between high-level abstraction (DQNN) and structured decision refinement (SpinalNet) by presenting hierarchical decision paths that mimic human diagnostic processes. The comparison between traditional methods and the proposed triple-way processing approach is summarized in Table 1.

**Table 1:** Comparison of traditional methods vs triple preprocessing.

Stage	Traditional methods	Triple preprocessing
Denoising	Gaussian/Median filters	**CEAMF**: Spatially adaptive filter that removes noise & artifacts contextually
Normalization	Min-max or histogram equalization	***Z*-score Normalization**: Standardizes image globally with statistical consistency
Contrast enhancement	Global contrast stretch	**Context-Aware Energy Mapping**: Uses spatial and pixel connectivity to enhance only diagnostically relevant regions

## 3 System architecture

**Figure 1:**
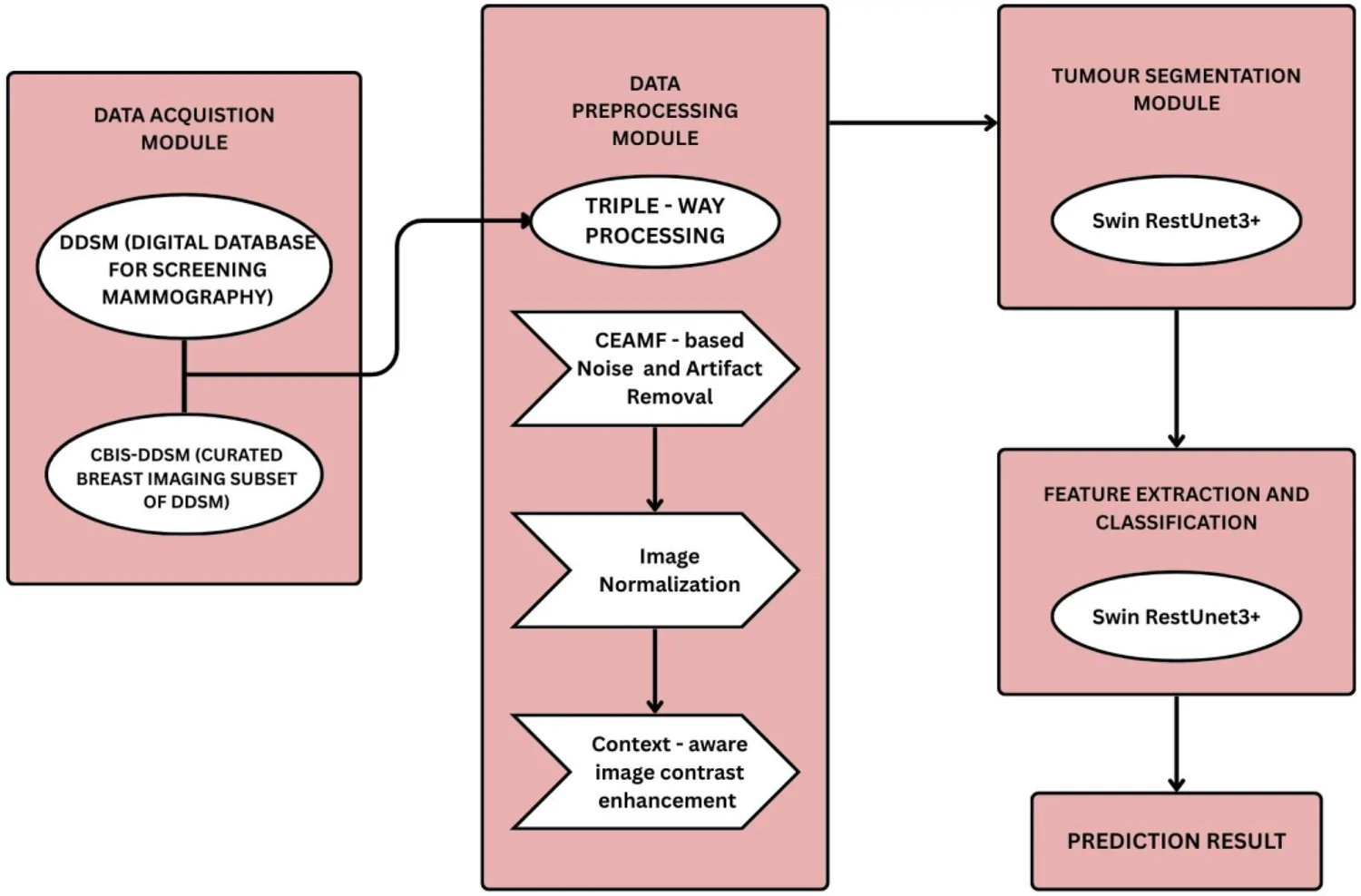
Q-SpinalNet-based breast cancer detection system.

The proposed system architecture, illustrated in Figure 1, begins with the Data Acquisition Module, where mammogram images are collected from the DDSM and its curated subset, CBIS-DDSM. These images are then transferred to the Data Preprocessing Module, which utilizes a three-stage approach consisting of CEAMF-based noise and artifact removal, image normalization, and context-aware contrast enhancement to improve image quality. The Tumour Segmentation and Classification Module utilizes the Swin RestUNet3+ model to achieve accurate tumor segmentation, feature extraction, and classification, leading to the final result.

Our proposed breast cancer detection system based on the Q-SpinalNet architecture is developed with mammography images obtained from two standard datasets, the DDSM (virtual Database for Screening Mammography) and the CBIS-DDSM (Curated Breast Imaging Subset of the DDSM). The diagnostic high-quality of the images is increased through a three-degree image preprocessing approach. Post-processing was conducted to erase the noise and artifacts of the CEAMF, whilst preserving all the requisite tissues. Delay side-by-side additions, image normalization (to establish a uniform range across all images), and context-automatic evaluation enhancements (to improve diffused pathological functions) were all utilized. The generated images were passed through the Swin ResUnet3+ model for accurate tumor segmentation. The ResUnet3+ was used as the decoder in the model to obtain local spatial information, and the Swin Transformer was used as the encoder to perform global contextual operations. Both Q-SpinalNet layer and Deep Quantum Neural network (DQNN) are hybrid class architectures; both receive the segmented tumor region. The DQNN employs quantum-inspired learning techniques with the help of superposition and probabilistic inference to achieve robustness and generalization. The Q-SpinalNet model processes input like the human spinal cord, with sequential inference using local and global functions. In DQNN, multiple possibilities are encoded to replicate the probabilistic nature of decision-making in the human brain. Inspired by the signal processing of spinal reflexes in the human spinal cord, SpinalNet integrates local feature extraction, hierarchical decision integration, and final decision making into an overall process. The output layer is the nucleus-type label indicating the class and type of breast cancer (benign or malignant) with a high degree of accuracy and low complexity. The model offers a thorough architecture for early and reliable detection of breast cancer from mammogram images.

## 4 Proposed system

### 4.1 Data acquisition

The images used in this study were obtained from two established mammography datasets: the Digital Database for Screening Mammography (DDSM) and the Curated Breast Imaging Subset of DDSM (CBIS-DDSM) (28). The DDSM data is a vastly superior validation dataset consisting of 13,128 images (5,970 benign and 7,158 malignant) that spans more than 50 institutions and includes rich medical information, including demographics of the affected persons and the interpretation of mammograms. The dataset is critical to the learning and testing of deep learning models that are intended to enhance the accuracy of breast cancer screening. The CBIS-DDSM dataset is a stronger and better-edited version of the DDSM, which enables the additional development of research in CAD. It carries images of mammography with ordinary, benign, and malignant tissues, all confirmed by pathological diagnosis. The data consists of 10,239 images consisting of 753 calcification cases and 891 loads cases. Qualified mammographers select those images and then they are decompressed and converted into DICOM format. Specifically, CBIS-DDSM focuses on hundreds of calcifications, which is a good basis of training, and comparing CAD models. For consistency, all are resized to 1,024 × 1,024 pixels. The preprocessing pipeline incorporates normalization to standardize pixel intensities, image filtering to minimize noise, and cropping to emphasize regions of interest. To strengthen model robustness and avoid overfitting, statistics augmentation strategies like flipping, zooming, and uniform resizing are carried out. Moreover, QOLCT-based feature extraction is utilized due to its robustness against fine fluctuations. The aggregate of statistical and form-primarily based capabilities, alongside normalization and regularization, guarantees that the version stays resilient throughout various datasets, allowing dependable and effective breast cancer detection. The datasets are divided into 75% for training, 12% for validation, and 13% for testing, with a balanced distribution of benign (60%) and malignant (40%) cases throughout all subsets to guarantee constant and reliable version evaluation. Table 2 provides the dataset details.

**Table 2:** Dataset details.

Dataset	Imaging modality	Total images	Class distribution	Key characteristics	Preprocessing & split
DDSM	Mammography	13,128	Benign: 5,970 (60%) Malignant: 7,158 (40%)	Collected from over 50 institutions; includes demographic and diagnostic metadata; highly suitable for validation of deep learning-based CAD systems.	Images resized to 1,024 × 1,024; normalization, noise filtering, and ROI cropping applied; data split into 75% training, 12% validation, and 13% testing.
CBIS-DDSM	Mammography	10,239	Calcification cases: 753 Mass cases: 891 Benign and Malignant (pathologically confirmed)	Curated and standardized version of DDSM; expert-annotated by certified mammographers; images provided in DICOM format; focused on masses and calcifications.	Resized to 1,024 × 1,024; normalization, denoising, cropping, and augmentation (flipping, zooming, resizing); QOLCT-based feature extraction; 75% training, 12% validation, 13% testing.

### 4.2 Data pre-processing

#### 4.2.1 Triple-way image preprocessing

**Figure 2:**
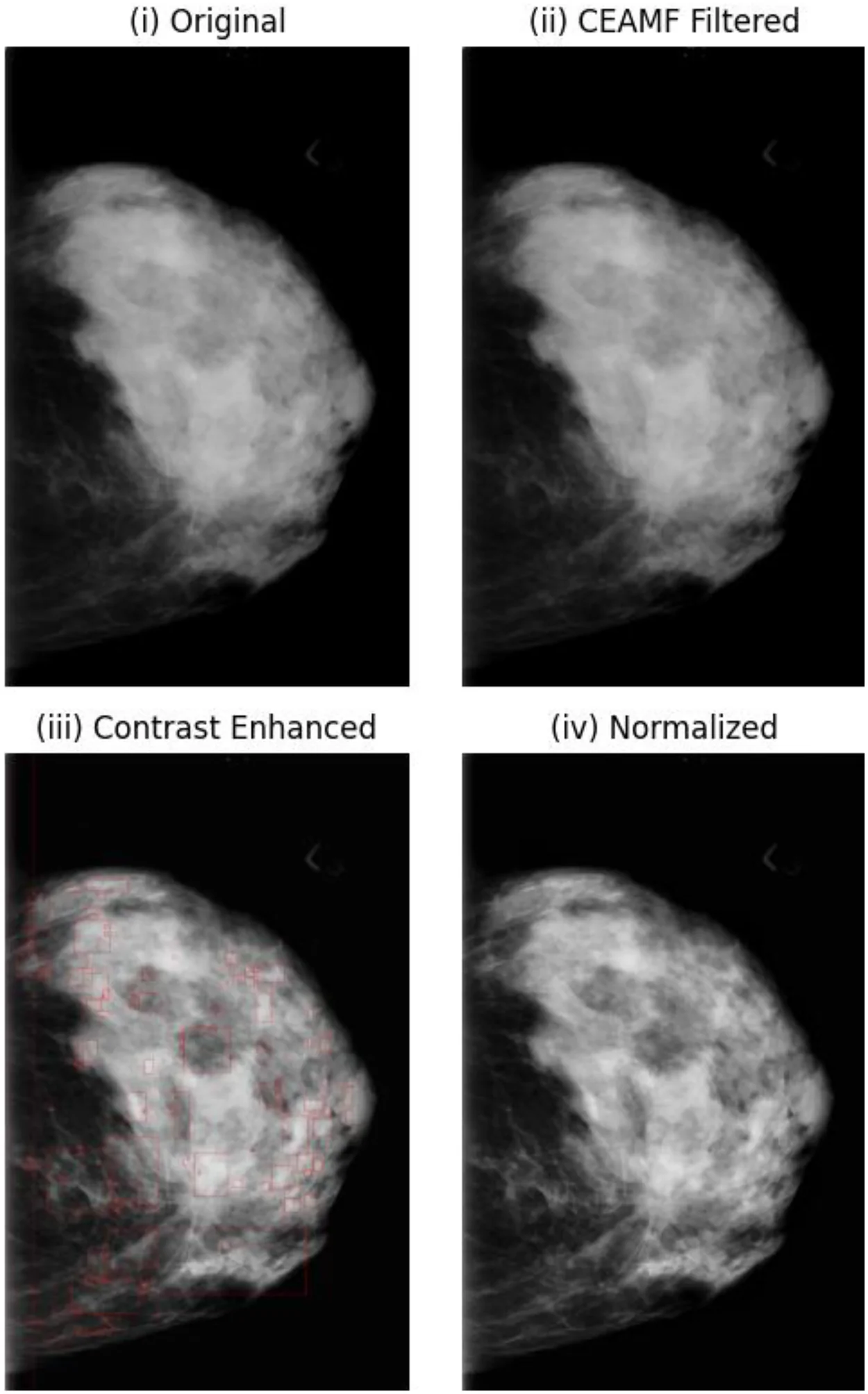
Triple-way image preprocessing: **(i)** input image **(ii)** CEAMF-based elimination of unwanted noise and artifacts, **(iii)** context-aware contrast enhancement, and **(iv)** image normalization.

The mammogram images obtained from the datasets often exhibit subpar quality, frequently degraded by noise, artifacts, and low contrast.To alleviate the demands at the classifier and improve the accuracy of most cancer analysis, suitable preprocessing is vital. In this study, preprocessing is carried out in three key ways: (i) CEAMF-based elimination of unwanted noise and artifacts, (ii) context-aware contrast enhancement, and (iii) image normalization. Images with applied Triple–way image processing are given in Figure 2 with input images.

#### 4.2.2 Ceamf-based noise and artifact elimination

To address source-specific noise and acquisition artifacts, preprocessing steps were applied separately to each dataset in the first stage:

- **Noise suppression:** To minimize high-frequency sensor noise while maintaining edge information, adaptive filtering (median/Gaussian, dataset-dependent) was used.
- **Artifact correction:** To reduce scanner-induced intensity inhomogeneities in imaging data, bias field correction and contrast normalization were used. For non-imaging datasets, missing values and outliers were handled using statistically bounded imputation.
- **Resolution and format standardization:** To facilitate downstream fusion, all samples were resampled to a uniform spatial or feature resolution and transformed into a common data representation.

Before cross-dataset integration, this step makes sure that every dataset is internally consistent.

##### 4.2.2.1 Stage II: feature alignment and cross-dataset normalization

To overcome inter-source variability, the second step concentrates on harmonizing data distributions across datasets:

- **Global normalization:** To mitigate distributional shifts, intensity or feature scaling (z-score/min–max normalization) was implemented using statistics calculated at the dataset level.
- **Feature alignment:** Across datasets, only common and semantically consistent features were kept. Features unique to each dataset were either mapped onto a common latent form or eliminated.
- **Verification of label consistency:** To prevent label ambiguity during training, annotation formats and class definitions were standardized. The learning model may generalize across diverse sources thanks to this stage, which also lessens domain bias.

##### 4.2.2.2 Stage III: model-ready transformation and task-aware improvement

Preprocessing in the last phase is customized to the learning goal:

- **Dimensionality refinement:** Task-relevant feature selection was used, and redundant or low-variance features were eliminated.
- **Data balancing and augmentation:** Controlled augmentation and resampling techniques were used to address sampling bias and class imbalance.
- **Model-ready encoding:** To ensure compliance with the learning process, data were encoded into the final form needed by the proposed design.
  This step guarantees that the input data is optimized for the downstream model in addition to being clean.

#### 4.2.3 Implementation consistency

To guarantee repeatability, all three steps were administered in a predetermined sequential order, and the same parameter values were kept constant across the trials. Crucially, pretreatment parameter estimates avoided data leakage by not using any information from test or validation sets.

The primary objective of this stage is to remove artifacts and noise from mammogram images to enhance diagnostic clarity.Common artifacts include background interference, opacity, external objects placed on the breast, motion-related distortions, embedded data, and machine-induced artifacts. The noise types addressed are Gaussian, speckle, salt-and-pepper, quantum, and impulse noise. To tackle this, the proposed CEAMF filter scans each pixel of the image and removes unwanted noise and artifacts. Let the input image be denoted as y, with dimensions Z × M, where y_ji denotes the pixel position of the image (j,i) where (j,i) ∈ B = {1,…,Z} × {1,…,M}. Since a larger filtering size increases computation time, a size limit **U** **×** **U** is applied. The maximum allowable size **U** is defined as

$$U=\frac{\min1\leq k\leq K(\min(hek,wik))}{4}$$ (1)

Here, hek and wik are the height and width of the light spot at index k, x is the total number of such spots being considered, and *U* is the upper limit size. The lower limit size for the CEAMF was initialized because using a smaller size did not yield effective noise and artifact elimination. It was ensured that this lower limit would not be less than 3 × 3. The corresponding size of **U** is defined by Equation 2.

$$U=\min\left(2\left(e^{\frac{a_{\mu }^{near(y)}}{sdis_{yt}^{near(y)}}}-1\right)+3,Sdis\right)$$ (2)

In this equation, **dsis^(near)^_(ji)_** represents the distance from the center of the light spot (i.e., noise or artifact) to the boundary of the noisy/artifact region. This distance, **dsis^(near)^_(ji)_(y)**, is further calculated using Equation 3.

$$sdis_{\;ji}^{near}(y)=\frac{{\left(\;j-cen_{y}^{near}\right)}^{2}+wi_{near}^{2}}{4{\left(i-cen_{x}^{near}\right)}^{2}}$$ (3)

Let $cen_{y}^{near}\;$ and $cen_{x}^{near}$ represent the center coordinates of the light spot, and let (j,i) denote the pixel coordinates. If the current pixel lies within the light spot's frame, the value of U can be computed using Equation 4:

$$U=\min\left(2\left(log_{10\left(\frac{sdis_{\;ji}^{near(y)}}{a_{\;ji}^{near(y)}}\right)}\right)+3,Sdis\right)$$ (4)

Assuming a window of size U x U is centered at pixel \((j, i)\), the CEAMF window set \(y_{j,i}(U)\) is defined by Equation 5:

$$y_{j,i}(U)=\left\{(q,w):|q-j|\leq \frac{U-1}{2},|w-i|\leq \frac{U-1}{2},(q,w)\in B\right\}$$ (5)

From this, the median value of the pixels in the window is denoted as $y_{j,i}^{\mathrm{medi}}(U)$. Accordingly, the CEAMF filtering operation is described in Equation 6:

$$y_{j,i}=y_{j,i}^{\mathrm{medi}}(U)$$ (6)

After applying this filter, the image can be reconstructed to remove noise and artifacts. The detailed steps are provided in Algorithm 1.

Algorithm 1 **Noise and Artifact Removal Using CEAMF.**

1. Input: The dataset's image y

2. Output: An image devoid of artefacts and noise

2. Begin

4. For every pixel (j,i)∈B do:

5.  Initialize U = 3

6.  Compute $sdis_{\;ji}^{near}(y),a_{\;ji}^{near}(y)$ and $y_{j,i}^{\mathrm{medi}}(U)$

7.  If $sdis_{\;ji}^{near}(y)<a_{\;ji}^{near}(y)$ then:

8.   Update U using:

       $U=\min\left(2\left(e^{\frac{a_{\mu }^{near(y)}}{sdis_{yt}^{near(y)}}}-1\right)+3,Sdis\right)$

9.  Else:

10.   Update U using:

      $U=\min\left(2\left(log_{10\left(\frac{sdis_{\;ji}^{near(y)}}{a_{\;ji}^{near(y)}}\right)}\right)+3,Sdis\right)$

11.  Apply CEAMF filter:

    $y_{j,i}=y_{j,i}^{\mathrm{medi}}(U)\;\;\;\;\;$

12.  End if

13. End for

14. End

#### 4.1.2 Image normalization

To enhance the differentiation between various image regions—such as distinguishing between low- and high-intensity areas—the preprocessed image is normalized. Let yi (where i = 1,2,…,N) depict the image with N pixels that has been denoised. The standard deviation and mean of the image are calculated using the following Equation 7:

$$\nabla_{y}=\sqrt{\frac{1}{N}}\overset{N}{\underset{i=1}{\sum }}\;(y_{i}-\partial_{y}{)}^{2}$$ (7)

Here, Δyis the standard deviation, and ∂y, the mean of the image, is defined using Equation 8:

$$\partial_{y}=\frac{1}{N}\overset{N}{\underset{i=1}{\sum }}\;y_{i\;}$$ (8)

Equation 9 can be used to formulate *Z*-score normalisation using the mean and standard deviation:

$$y^{\mathrm{Zs}}=\mathrm{Zs}(y)=\frac{y-\partial_{y}1}{\forall_{y}}$$ (9)

In this equation, 1 denotes a vector of ones [1,1,…,1]^T, indicating that every pixel in the noise/artifact-reduced image is normalized to a scale relative to 1.

#### 4.1.3 Context-based image contrast improvement

To increase the image's visual quality, contrast enhancement is applied after normalization, especially by boosting contrast within particular intensity ranges. The suggested technique modifies the energy distribution of the image by taking into account its context. The image's energy curve is obtained using a neural network that treats each neuron as a pixel. This method guarantees that the contrast is adjusted according to the content of the image.

Weights connect each pixel to its closest neighbors; a value of “1” signifies a connection, while “0” signifies no connection. Examine an image with dimensions Z × M, where R is the total number of grey levels and pix_ij_ is the intensity value of the pixel at location (i, j) in a grayscale range of [0, R − 1]. The set of nearby pixels surrounding (i, j) within a given distance d is referred to by the term $M_{\mathrm{ij}}^{d}$. Equation 10 defines the image's energy curve using this configuration:

$${\mathrm{ene}}_{in}^{f}=-\overset{2}{\underset{j=1}{\sum }}\;\overset{M}{\underset{j=1}{\sum }}\;\underset{{\mathrm{ghem}}_{\mathrm{ij}}^{d}}{\sum }\;\mathrm{pi}x_{\mathrm{ij}}\cdot \mathrm{pi}x_{\mathrm{gh}}+\overset{2}{\underset{i=1}{\sum }}\;\overset{M}{\underset{j=1}{\sum }}\;\underset{\mathrm{gh}\in M_{\mathrm{ij}}^{d}}{\sum }\;C_{\mathrm{ij}}\cdot C_{\mathrm{gh}}$$ (10)

The spatial correlation matrix is represented by C_ij_, the grey level is indicated by (in), and f is a variable coefficient that ranges from 1 to 3. A clipping procedure is used to regulate the enhancement rate once the energy curve has been computed. Equation 11 provides the clipped energy curve:

$$\mathrm{Ene}\;(\mathrm{cl})=\{\begin{array}{c} C_{\mathrm{clipp}\;},\;\mathrm{ifEne}\;(cl)\geq C_{\mathrm{clipp}\;}\\ \;\mathrm{Ene}\;(\mathrm{cl}),\;\mathrm{Otherwise}\; \end{array}$$ (11)

Here, Cclipp is the clipping threshold used in the clipping operation.

- Ene(cl) is the energy curve after clipping,
- Cmean is the average spatial correlation across all pixels in the image, and
- Cmedian is the median of the spatial correlation values.

To categorize the energy curve into three different regions, the standard deviation (Std) is calculated using the formula provided in Equation 12:

$$\mathrm{StD}=\sqrt{\left(\frac{\sum_{0}^{R-1}\;{\left(r-\;\mathrm{ene}{\;}_{\mathrm{mean}\;}\right)}^{2}\times \;\mathrm{ene}\;(\mathrm{cl})}{\sum_{r=0}^{R-1}\;\mathrm{ene}(\mathrm{cl})}\right)\;}$$ (12)

Where:

- enemean represents the average value of the clipped energy curve.
- Based on these values, upper (LO_high_) and lower (\(LO_ {low}\)) thresholds for the energy curve are established, as defined in Equations 13, 14.
  $$Lo_{\mathrm{low}\;}=L_{0}+StD$$ (13)
  $$L_{\mathrm{Ohigh}\;}=L_{R-1}+StD$$ (14)

Equations 13, 14 indicate that the image's minimum and maximum intensity levels are represented by $L_{0}$ and $L_{R-1}$, respectively. The energy curve is separated into three separate supplemental bands based on these intensity values: from 0 to L_low_, from L_low_ + 1 to L_high_, and from L_high_ + 1 to R − 1. Each of these supplementary energy curves is processed independently to generate the final transfer function. This procedure consists of three main stages: calculating the probability density function (pdf), obtaining the cumulative distribution function (cdf) from the pdf, and creating the transfer function (Trf). The possibility density capabilities for the three extra strength ranges are represented through Equations 15–17.

$$\mathrm{pd}f_{\mathrm{lo}}(l)=\;\mathrm{ene}\;(l)/p_{\mathrm{lo}}\;\mathrm{for}\;0\leq l\leq Lo_{\mathrm{low}\;}$$ (15)

$$\mathrm{pd}f_{\mathrm{me}}(l)=\;\mathrm{ene}\;(l)/p_{\mathrm{me}}\;\mathrm{for}\;Lo_{\mathrm{low}\;}+1\leq 1\leq Lo_{\mathrm{high}\;}$$ (16)

$$\mathrm{pd}f_{\mathrm{up}\;}(l)=ene(l)/p_{\mathrm{up}}\;\mathrm{for}\;Lo_{\mathrm{high}\;}+1\leq 1\leq R-1$$ (17)

According to these equations, the full electricity levels for the three additional curves are indicated as p_lo_, p_me_, and p_high_, respectively. The related cumulative distribution features (cdfs) are described using Equations 18–20.

$$\mathrm{cd}f_{\mathrm{lo}}(l)=\overset{1}{\underset{k=0}{\sum }}\;\;\mathrm{pdf}{\;}_{\mathrm{lo}}(k)\;\mathrm{for}\;0\leq 1\leq Lo_{\mathrm{low}}$$ (18)

$$\mathrm{cd}f_{\mathrm{me}}(1)=\overset{1}{\underset{k=\mathrm{Lolow}+1}{\sum }}\;\mathrm{pd}f_{\mathrm{me}}(k)\;\mathrm{for}\;Lo_{\mathrm{low}}+1\leq 1\leq LO_{\mathrm{high}\;}$$ (19)

$$\mathrm{cd}f_{\mathrm{up}}(1)=\overset{1}{\underset{k=\mathrm{Lohigh}+1}{\sum }}\;\mathrm{pd}f_{\mathrm{up}}(k)\;\mathrm{for}\;Lo_{\mathrm{high}}+1\leq 1\leq R-1L$$ (20)

Based on the cumulative distribution function (cdf), the transfer function for the supplementary energy curves can be defined using following Equations 21–23:

$$\mathrm{Tr}F_{\mathrm{lo}\;}=Lo_{\mathrm{low}\;}\times \mathrm{cd}f_{\mathrm{lo}\;}$$ (21)

$$\mathrm{Tr}F_{\mathrm{me}}=(Lo_{\mathrm{low}\;}+1)+(Lo_{\mathrm{high}\;}-Lo_{\mathrm{low}\;}+1)\times \mathrm{cd}f_{\mathrm{me}}$$ (22)

$$\mathrm{Tr}F_{\mathrm{up}}=(Lo_{\mathrm{high}\;}+1)+(R-Lo_{\mathrm{high}\;}+1)\times \mathrm{cd}f_{\mathrm{up}\;}$$ (23)

The complete transfer function for image contrast enhancement is expressed in Equation 24:

TrF=TrFlo+TrFme+TrFhigh (24)

In this equation

- TrFlo represents the lower limit transfer function,
- TrFme refers to the middle limit transfer function, and
- TrFhigh denotes the transfer function for upper limit.

After preprocessing, a block-based 2D-to-3D conversion technique is used to convert the image into a 3D representation. Algorithm 2 below outlines the precise procedures for carrying out context-aware contrast augmentation.

Algorithm 2 **Context-based contrast improvement.**

Input: Image Normalisation (y^Zs^)

Output: Contrast Enhanced Function (TrF)

Steps:

1. Start the algorithm.

2. Initialize the image energy curve.

3. Compute the energy curve enecu using a weight function over [0, 1], where the input intensity range is [0, R − 1][0, R − 1].

4. Calculate the energy index enei using Equation 10.

5. Adjust the enhancement rate based on the energy curve, Ene(cl).

6. If the enhancement level Ene(l) is greater than or equal to the clipping threshold Cclipp:

 a. Clip the value using Cclipp=(Cmean + Cmedian)

7. Else:

 a. Clip using Ene(cl).

8. Split the energy curve enecu {ene}_{cu} based on Equations 12–14.

9. Initialize the final transfer function (TrF).

10. Compute the probability density functions (pdf) using Equations 15–17.

11. Derive the cumulative distribution functions (cdf) using Equations 18–20.

12. Generate the transfer function (TrF) using the cdf.

13. From the cdf, compute the supplementary energy curves using Equations 21–23.

14. Obtain the final transfer function using Equation 24.

15. End the algorithm

### 4.3 Tumor segmentation using Swin ResUnet3+

The collected image Ba is fed into the Swin ResUNet3**+** model for image segmentation within the proposed framework. There are three modules in the ResUnet3+. These are shallow feature extraction, ResUnet3+ feature extraction, and the reconstruction phase. These phases' specifics are listed below. For the shallow feature extraction phase, I and X represent the corrupted image resolution for the noisy input image Z∈S^I^ ^×^ ^X^ ^×^ ^3^. This model uses a single 3 × 3 convolution layer NSF() to extract low-frequency data, including the input image's texture or color. Equation 25 created B_shlw_ ∈ S^I×X×DB^, a shallow feature.

$$B_{\mathrm{shlw}}\;=\;N_{\mathrm{SF}}(Z)$$ (25)

In this case, the number of shallow feature channels is indicated by the variable D. The mode of feature extraction for ResUnet3+ uses the multi-scale and high-level deep features B_deep_ ∈ S^I×X×DB^, which are then retrieved using the ResUnet3+ feature extraction NUF(.) using the shallow feature B_shlw_.

**Reconstruction phase:** Finally, the 3 × 3 convolution layer NR(.) uses the deep features B_deep_ which are stated in Equation 26, to produce the noise-free image Z^∈N_R_(B_deep_)

$$Z^{\wedge }=\mathrm{NR}(\mathrm{Bdeep})$$ (26)

Using the sound image Z as the input of ResUnet3+, the term z ^ is obtained. It is the image Z's clean version and ground truth. “STL” is a multiple of two, with one representing “Window Multi-head Self-Attention (W-MSA)” and the second representing “Shifted-Window Multi-head Self-Attention (SW-MSA).” Equations 27–30 provide the swin transformer capabilities.

$${\hat{g}}^{M}=X-MSA(L\mathrm{Nor}(g^{M-1}))+g^{M-1}$$ (27)

$$g^{M}=MLP(\mathrm{LNor}({\hat{g}}^{M}))+{\hat{g}}^{M}$$ (28)

$${\hat{g}}^{M+1}=SW-MSA(\mathrm{LNor}(g^{M}))+g^{M}$$ (29)

$$g^{M+1}=MLP(\mathrm{LNor}({\hat{g}}^{M+1}))+{\hat{g}}^{M+1}$$ (30)

In these equations, *LNor* represents Layer Normalization, and *MLP* refers to Multi-Layer Perceptron. The self-attention mechanism used in the model is defined in Equation 9:

$$\mathrm{Attention}\;(I,X,D)=\mathrm{softmax}\left(\frac{IX^{U}}{\sqrt{e}}+C\right)D$$ (31)

**Figure 3:**
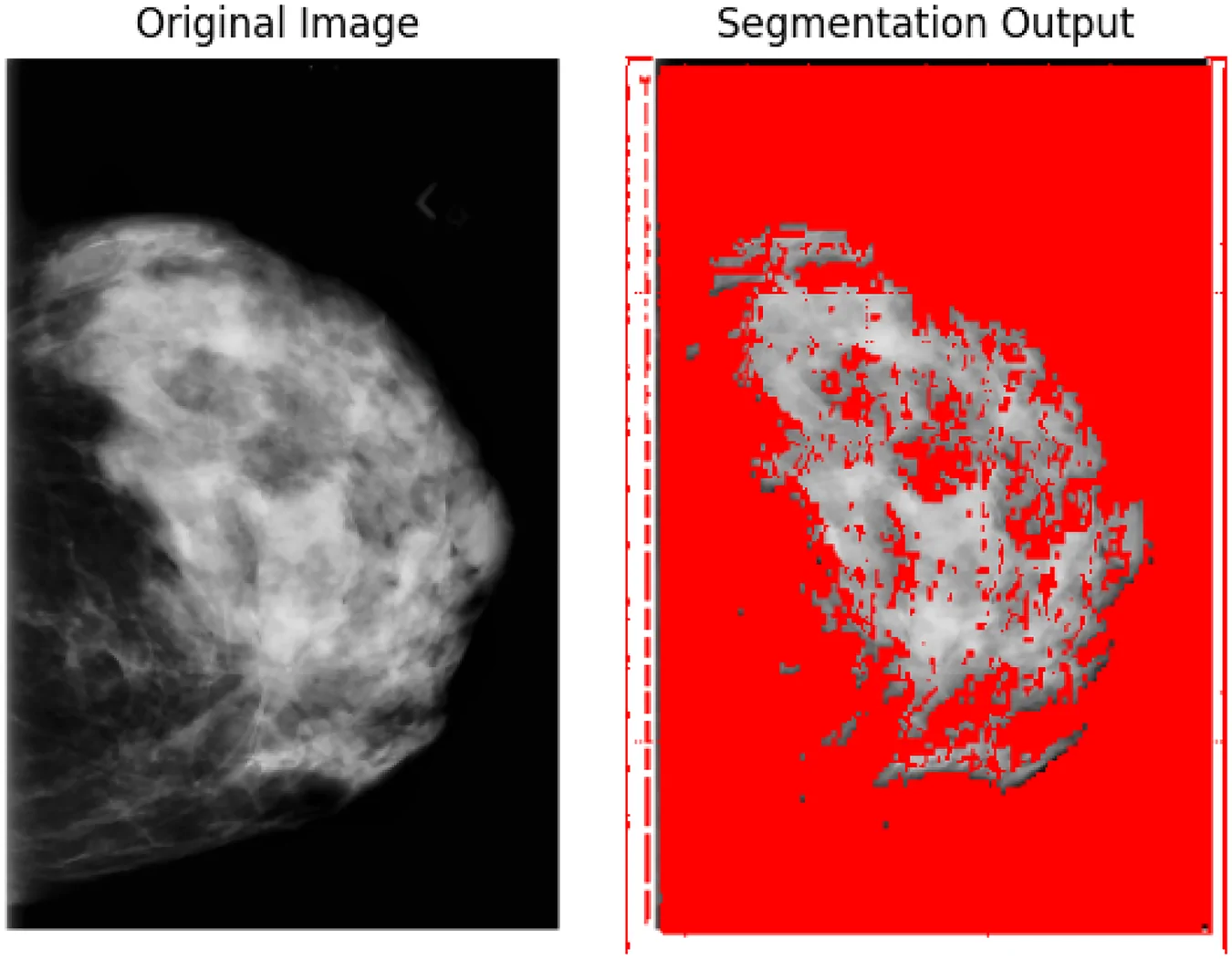
Swin ResUnet3+ segmentation image output compared to the original image.

Here, *C* denotes the bias matrix. The segmented outputs Bseg_a produced by the Swin ResUnet3+ model are gathered and used for subsequent processing, as illustrated in Figure 3.

### 4.4 BC detection using Q-SpinalNet

The incidence of BC (Breast Cancer) is rising in developing countries, primarily due to increased life expectancy and changes in lifestyle. Detecting BC is essential since it aids in identifying genetic characteristics linked to cancer and forecasting the course of specific illnesses. Numerous techniques have been put forth for BC pattern classification and prediction. In this respect, Q-SpinalNet is a unique method for BC detection that combines the characteristics of DQNN and SpinalNet.

**Figure 4:**
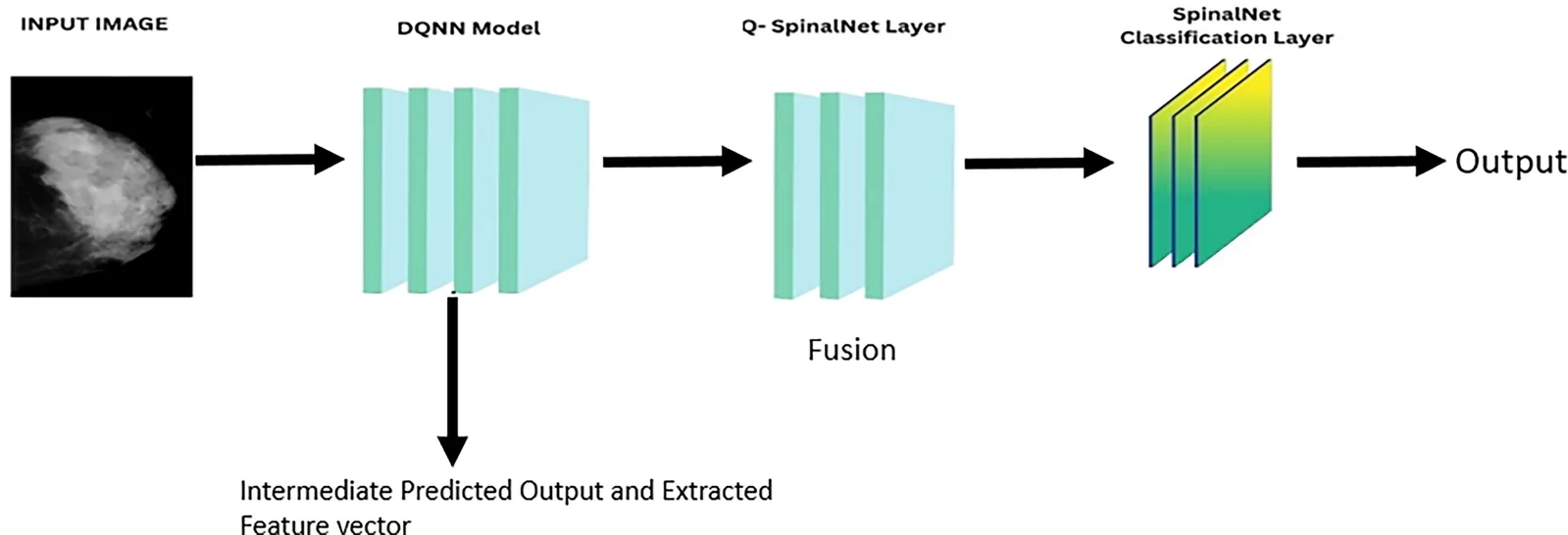
BC detection using Q-SpinalNet.

The DQNN model is first fed the input mammographic image. The Q-SpinalNet layer receives both the extracted feature vector and the output produced by the DQNN model. Fusion and regression procedures are used in this layer to calculate how similar the actual and expected outputs are. The final BC detection result is obtained by feeding the output from the Q-SpinalNet layer into the SpinalNet model. The BC Detection Using Q-SpinalNet is shown in Figure 4.

#### 4.4.1 DQNN model

Using entire positive layer transition maps, the DQNN (44) model is a quantum equivalent of conventional back-propagation methods. The DQNN processes an input MG image Mb. The quantum perceptron, a quantum variant of the conventional perceptron used in machine learning, is the central component of DQNN. The input qubits (u) and output qubits (v) are managed by these quantum perceptrons, which work as random unitary functions. Under the control of (2u + v)^2 − 1 parameters, each perceptron manages both u + v input and output qubits. Input qubits are initialized using potentially unknown configurations *η*^in^, while output qubits start in a default product state ∣0…0⟩_out_. The model simplifies calculations using a (*u* + 1)-qubit unitary operator.

The overall DQNN architecture is defined through quantum neurons. These neurons are structured in a quantum circuit where quantum perceptrons are arranged across *D* hidden layers. Initially, the circuit is fed with input qubits configured as *η*^in^, and the processing leads to output qubits characterized by *η*^out^. The final output state is given by Equation 32:

$$\eta^{\mathrm{out}\;}=tr_{\mathrm{in},h\;}\left(ℜ\left(\eta^{\mathrm{in}\;}⊗\left|{0\ldots 0\rangle }_{h,out}\langle 0\ldots 0\right|\right){ℜ}^{*}\right)$$ (32)

Quantum perceptron product operations on the qubits in layers *τ* and *τ*−1 are represented by layer unitaries denoted by R*τ*. Broad quantum prediction using both one- output and dual-input qubit perceptrons is a direct result of quantum circuit arrangement; the DQNN's network output is a crucial aspect. It is described as a sequence of completely positive layer-to-layer transition maps, denoted as x*τ*:

U_b=χ_out(χ_T(…χ_2(χ_1(h_in))…)) (33)

where $x_{\tau }(z_{\tau -1})=tr_{\tau -1}(\sum_{j=1}^{m_{\tau }}R_{\tau j}(z_{\tau -1}))/(\sum_{j=1}^{m_{\tau }}R_{\tau j})$.

Here, $R_{\tau j}$ refers to the perceptron operating between layers τ and τ−1.

The input state is defined by η_in, and the total number of perceptrons at layer τ is represented by the parameter $m_{\tau }$. Critical structural behavior is reflected in the DQNN output, which is crucial for creating quantum counterparts of traditional back-propagation techniques.

Furthermore, the DQNN satisfies quantum perceptron requirements through the use of managed unitary operations, formulated using Equation 34:

$$ℜ=\underset{\zeta }{\sum }\;\left|\zeta \rangle \langle \zeta \right|⊗ℜ(\zeta )$$ (34)

In this expression, ζ denotes the index over the basis states, R(ζ) represents the parameterized unitary operator, and |ζ⟩ denotes the basis vectors in the input space. The output condition is derived from measurement results and is expressed using Equation 35:

$$\eta^{\mathrm{out}\;}=\underset{\zeta }{\sum }\;\langle \zeta \left|\eta^{\mathrm{in}\;}|\zeta \rangle ℜ(\zeta )|0\rangle \langle 0\right|ℜ(\zeta {)}^{*}$$ (35)

It is important to note that the channels in question do not rely on typical quantum evaluations and do not have non-zero quantum capacity.

#### 4.4.2 Q-SpinalNet layer

Ab is defined as follows and is used as the input for the fusion and regression procedures within the Q-SpinalNet layer:

$$A_{b}\;=\;\{\;U_{b,}V_{b}\}$$ (36)

Here, V_b_ represents the feature vector, while U_b_ indicates the output from the DQNN. The following sections describe the expressions used in the Q-SpinalNet layer.

$$p=\overset{5}{\underset{r=1}{\sum }}\;V_{1T}*Wr$$ (37)

$$p_{1}=\underset{T=1}{\sum }\;x\underset{J=1}{\sum }\;zV_{2\tau \gamma }*W_{\tau ,\beta }$$ (38)

$$p_{2}=\underset{T=1}{\sum }\;x\underset{J=1}{\sum }\;zV_{3rr}*W_{r,\xi }$$ (39)

$$p_{3}=\underset{T=1}{\sum }\;x\underset{J=1}{\sum }\;zV_{4\tau r}*W_{r,\xi }$$ (40)

$$p_{4}=\underset{r=1}{\sum }\;x\underset{J=1}{\sum }\;zV_{5\pi \tau }*W_{r,\pi }$$ (41)

Where p, p1, p2, p3, and p4p, represent the outputs from the t^th^, (t − 1)^th^, (t − 2)^th^, (t − 3)^th^ and (t − 4)^th^ intervals. The equation can be written as follows by using Fractional Calculus (FC) (41):

$$\begin{array}{rl} Q_{b}= & \mu *p+\frac{1}{2}\mu *p_{1}+\frac{1}{6}(1-\mu )p_{2}+\frac{1}{24}(1-\mu )(2-\mu )p_{3}\\ + & \frac{1}{120}(1-\mu )(2-\mu )(3-\mu )p_{4}\\ + & \frac{1}{720}(1-\mu )(2-\mu )(3-\mu )(4-\mu )p_{5} \end{array}$$ (42)

$$\begin{array}{rl} Q_{b}= & \mu *\overset{5}{\underset{\tau =1}{\sum }}\;V_{1r}*Wr+\frac{1}{2}\mu *\underset{\tau =1}{\sum }\;x\underset{J=1}{\sum }\;zV_{2r,s}*W_{r,s}\\ + & \frac{1}{6}(1-\mu )\underset{\tau =1}{\sum }\;X\underset{J=1}{\sum }\;zV_{3r,s}*W_{r,s}\\ + & \frac{1}{24}(1-\mu )(2-\mu )\underset{\tau =1}{\sum }\;X\underset{J=1}{\sum }\;zV_{4r,s}*\\ + & \frac{1}{120}(1-\mu )(2-\mu )(3-\mu )\underset{r=1}{\sum }\notin \underset{s=1}{\sum }\;zV_{5r,\xi }*W_{r,s}\\ + & \frac{1}{720}(1-\mu )(2-\mu )(3-\mu )(4-\mu )U_{b} \end{array}$$ (43)

#### 4.4.3 Spinalnet model

The SpinalNet model is inspired by the structural design of the human spinal cord and is applied to artificial neural networks (ANNs), hence the name SpinalNet. Qb is the input that the SpinalNet model uses.

Inputs are fed gradually over regular periods in this model. During processing, both local and global output components are taken into account. Every layer in the network produces a local output, and a global output is created using the input, which is amplified. The ANN weights are optimized using training data.

Each neural network (NN) layer in the classical SpinalNet (45) architecture is separated into three segments: input, intermediate, or output. A fraction of the entire input data is sent to each NN layer's input segment. Every intermediate segment, with the exception of the first layer's intermediate portion, receives two components: one from the input segment of the current layer and one from the preceding intermediate layer.

$$P_{b}=\sigma \left(\overset{K}{\underset{t=1}{\sum }}\;(\omega_{nt})\xi t+R_{n}^{kn\infty }\right)$$ (44)

In this case, *σ* is the activation function, K is the total number of neurons, *ω*mi is the weight, and *ξ*i is the input from the ith neuron.

The number of neurons, including those in the hidden layer, may vary depending on the architecture. To lower the computational cost of multiplications, however, the number of neurons in the intermediate layers and the input values allocated to each layer are decreased. Equation 45 provides the sigmoid function, which is utilized as an activation:

$$\sigma (\delta )=\frac{1}{1+\exp(-\delta )}$$ (45)

Every input feature influences the final output because SpinalNet processes inputs gradually and repeatedly. The output layer usually uses a linear (or identity) activation, which means the output is equal to the input without transformation; The ReLU function is defined using Equation 46.

ReLU(d)=max(0,d) (46)

Intermediate layers can use nonlinear activation functions like softmax, ReLU, or sigmoid.

The output from the SpinalNet model for BC detection is denoted as P_b_. Algorithm 3 provides the Q-SpinalNet training pipeline.

Algorithm 3 **Q-SpinalNet training pipeline.**

**Input:**

- X = {x₁, x₂, … , x_n_}: Preprocessed mammogram images
- Y = {y₁, y₂, … , y_n_}: Corresponding labels
- Learning rate *η*, Number of epochs E

**Output:**

- Trained model parameters W (DQNN + SpinalNet)

**Step 1: Initialization**

1. Initialize DQNN parameters (quantum layers and perceptrons)
2. Initialize SpinalNet segments (for segment-wise input processing)
3. Initialize Swin-ResUnet3+ (use pretrained weights if available)

**Step 2: Training Loop**

For each epoch from 1 to E:

 For each image-label pair (x_i_, y_i_) in (X, Y):

  a. **Segmentation:**

   S_i_ = SwinResUnet3Plus(x_i_)

  b. **DQNN Feature Extraction:**

   (U_i_, V_i_) = DQNN(S_i_)

    U_i_: Output, V_i_: Quantum feature vector

  c. **Q-SpinalNet Classification:**

   A_i_ = U_i_ ∪ V_i_

   ŷ_i_ = SpinalNet(A_i_)

  d. **Loss Calculation:**

   Loss_i_ = CrossEntropy(ŷ_i_, y_i_)

  e. **Backpropagation:**

   Update DQNN and SpinalNet weights using gradient descent:

   W ← W − *η* * ∇Loss_i_

**Step 3: Model Evaluation**

- Evaluate trained model on validation/test set using Accuracy, Precision, Sensitivity, Dice score, etc.

**Return:** Optimized weights W

#### 4.4.4 Overall method for BC identification using MG images

Identifying malignant regions in MG images (46) is the initial stage of the breast cancer detection process, facilitating early diagnosis and reducing mortality rates. The Non-Local Means (NLM) filter is used to pre-process an input MG image after it has been obtained from the database. ET-SegNet, which combines ET-Net and U-Net, is then used for segmentation. Next, during the feature extraction phase, several descriptors such as LTP, FLBP, LST, LBP, PhOG, and MBP are extracted. The final detection step is carried out using Q-SpinalNet, a novel model that integrates a Deep Quantum Neural Network (DQNN) with SpinalNet.

#### 4.4.5 End-to-end processing chain: explicit algorithmic separation

Two strictly consecutive, functionally separate processing chains make up the proposed framework. These chains are not interchangeable and have different roles, outputs, and methods.

##### 4.4.5.1 Chain-1: classical image conditioning and feature augmentation (pre-segmentation stage)

**Purpose:** To improve lesion representation and image quality before deep learning without generating final segmentation or classification results.

**Input:** Raw mammogram image III

**Processing Steps:**

1. **Noise Elimination:**
  Anatomical features are preserved when acquisition noise is suppressed via Non-Local Means (NLM) filtering.
2. **Enhancement of Contrast**
  By increasing the contrast between diseased and normal tissue, Entropy Thresholding (ET) enhances lesion appearance.
3. **Coarse Delineation of Regions:**
  Coarse candidate lesion areas produced by ET-SegNet are solely utilized for feature support and not for final segmentation.
4. **Manually Designed Feature Extraction:**
  An auxiliary feature vector, denoted as F_hc_, is constructed by extracting texture, intensity, and shape descriptors from the enhanced regions.

**Output:** Enhanced image I_enh_ and auxiliary feature set F_hc_

**Significant Limitation:** Chain-1 is never assessed on its own and does not carry out diagnostic segmentation or classification.

##### 4.4.5.2 Chain-2: final decision stage

Deep Transformer–Quantum Segmentation and Diagnosis

Final lesion segmentation and malignancy classification are the goals.

**Input:** Auxiliary features and improved image I_enh_ FHC

**Steps in Processing:**

5. **Transformer-Based Segmentation:** The final pixel-level lesion mask Mseg is generated by Swin ResUnet3+.
6. **Deep Feature Extraction and Fusion:** To create the unified representation Ffusion, deep features from Swin ResUnet3+ are combined with F_hc_
7. **Quantum-Inspired Sequential Classification:** Q-SpinalNet processes the fused representation, performing progressive decision refining and producing the malignancy probability P_mal_.

**Output:** Final lesion segmentation mask M_seg_ and diagnostic prediction P_mal_.

### 4.5 Integration of quantum-inspired modeling with spinalNet

The proposed scheme combines SpinalNet with a quantum-inspired neural model in a complementary and functionally unique way. By simulating intricate feature interactions via probabilistic amplitude encoding and unitary-like transformations, the quantum-inspired component improves feature representation. This makes it possible to describe several feature dependencies at once and creates uncertainty-aware representations, which are especially useful for capturing subtle and unclear lesion properties.

While expressive, quantum-inspired representations typically function as global transformations, which may limit the interpretability of how specific feature groups contribute to the final classification.SpinalNet is used as the foundation for decision-making in order to overcome this constraint. To carry out sequential decision refinement across spinal segments and analyze and understand intermediate decision states, SpinalNet breaks down the fused feature space into ordered subsets. The allocation of duties between the two halves creates a synergy: SpinalNet organizes the decision-making process, while the quantum-inspired model enhances what is represented. While later spinal segments gradually clarify ambiguous instances by incorporating collected information, early spinal segments record crude discriminative signals. By making decisions incrementally, resilience is increased and sensitivity to noise or erroneous correlations is decreased.

Interpretability wise, the probabilistic results of the quantum-inspired model provide calibrated confidence predictions, whereas SpinalNet allows tracing the decision formation through layers. This complementary behavior is also supported by empirical tests of ablation, which show greater variance in the case of the monolithic classifier placed in the place of SpinalNet and lower sensitivity in the case of the quantum-inspired part removed. The combined outcomes indicate that the joint execution of structured sequential decision-making and quantum-inspired representation learning works effectively to increase the performance but not individually.

### 4.6 Synergy between quantum-inspired modeling and SpinalNet

The combination of quantum-inspired modeling with SpinalNet, which jointly solve complementary issues in medical image classification, is a significant contribution of this study. A rich, uncertainty-aware feature representation that can simulate intricate and nuanced lesion patterns is provided by the quantum-inspired component. On their own, though, these depictions might not be accurate when making decisions.

By implementing a sequential and modular selection procedure that evaluates feature subsets gradually, SpinalNet lessens this constraint. The diagnostic judgment might change gradually thanks to this architecture, which is quite like clinical reasoning. This allows for the examination of individual feature groups' contributions as well as their confidence across spinal segments.

The integrated design shows that enhanced performance is due to their coordinated interaction rather than just advanced representation learning or classifier complexity. While SpinalNet guarantees stable, comprehensible, and reliable decision-making, the quantum-inspired approach improves representational expressiveness. The observed improvement in sensitivity has clear clinical consequences that go beyond numerical increases. Higher sensitivity in breast cancer screening results in a decreased false-negative rate, which is crucial for identifying lesions with hazy borders and early-stage cancers. The proposed strategy obtains the best sensitivity, as seen in Figure 7, demonstrating increased dependability in detecting cancerous instances that might have gone undiscovered. This improvement validates its prospective use as a clinical decision-support tool, when the risk of overlooking a malignant lesion is much higher than that of producing more follow-up tests.

### 4.7 Interpretability of the quantum-inspired decision model

#### 4.7.1 Interpretability challenges in quantum-inspired models

By encoding features within a probabilistic amplitude space, quantum-inspired neural models offer enhanced representational capacity. However, because decision limits are created by global feature changes that are not immediately interpretable by humans, this strength also presents interpretability issues. Such “black-box” conduct may hinder adoption and trust in healthcare facilities.

#### 4.7.2 Quantum-inspired component: A behavioral interpretation

The proposed model's quantum-inspired module displays observable and comprehensible behaviors even if it does not rely on actual quantum hardware:

##### 4.7.2.1 Amplitude distributions as saliency of features

Probabilistic relevance weights are correlated with feature amplitudes. It is possible to identify dominating characteristics that contribute to classification confidence by tracking amplitude change across layers.

##### 4.7.2.2 Encoding uncertainty

Prediction confidence is reflected in the probabilistic output distribution. Ambiguous instances are associated with broader distributions, which is consistent with clinically challenging lesions.

##### 4.7.2.3 Perturbation-resistant stability

Stable decision generation as opposed to fragile black-box behavior is indicated by controlled perturbation of input characteristics, which results in incremental decision modifications rather than sudden flips.

#### 4.7.3 Spinalnet as an interpretability scaffold

SpinalNet is strategically integrated to structure and elucidate the quantum-inspired decision process. A partial quantum-encoded feature subset is sent to each spinal segment, which generates an intermediate prediction. This makes it possible to

- Refine decisions by sequential scrutiny
- Determine which feature groups affect early vs. late phases of decision-making and provide confidence across visualized spinal segments
- Carry out clinical diagnostic procedures, where evidence is gathered rather than instantly assessed, which is similar to this progressive reasoning pathway.

## 5 Performance metrics

### 5.1 Performance metrics in comparison with existing algorithms

**Figure 5:**
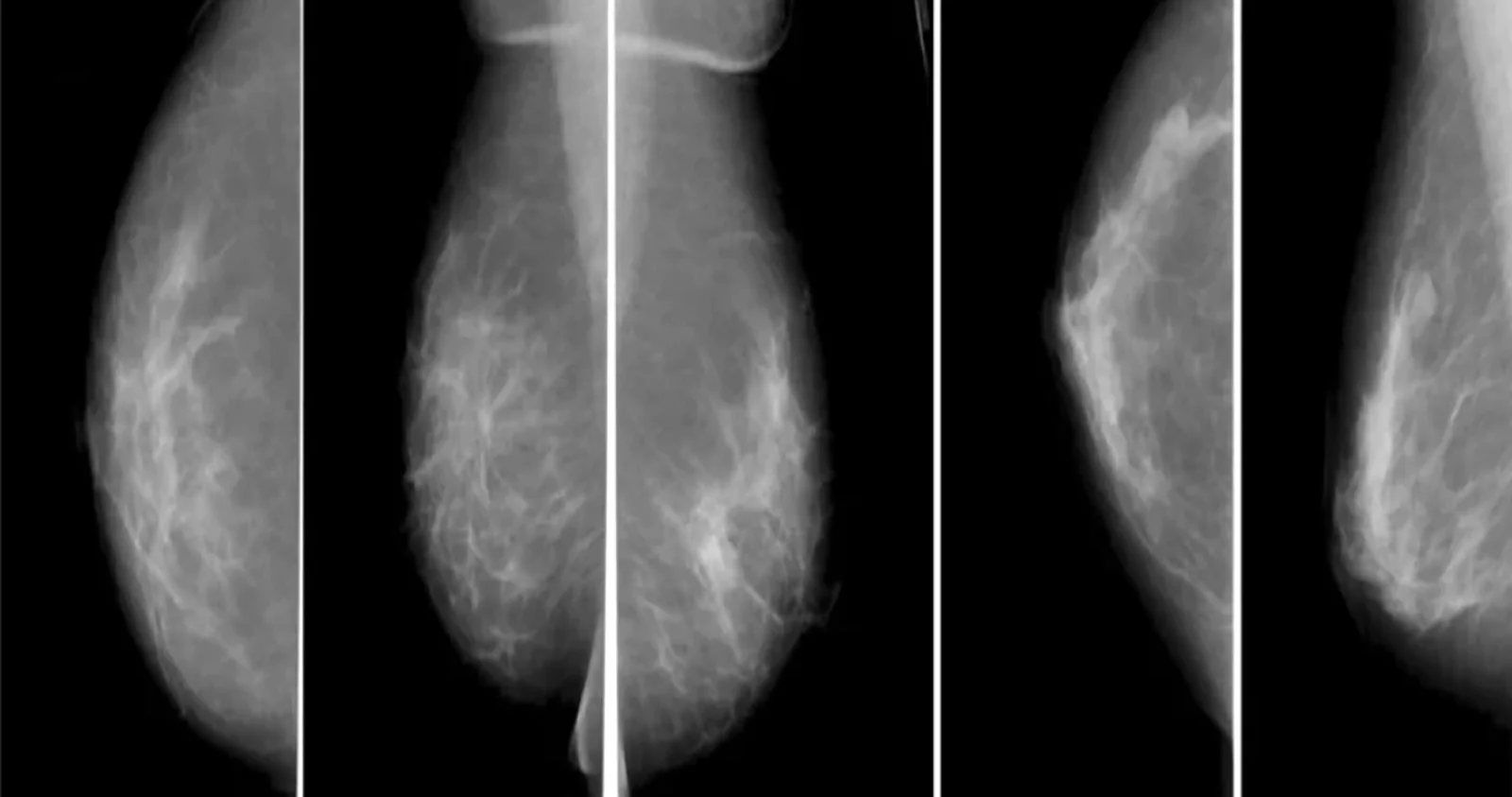
Sample dataset images.

The performance of the proposed Deep Quantum Neural Network combined with Swin ResUnet3+ for breast tumor segmentation and classification is evaluated using a comprehensive set of standard metrics. These metrics are designed to assess lesion detection accuracy, segmentation precision, and classification robustness, particularly important when working with imbalanced medical imaging datasets such as DDSM and CBIS-DDSM (47). Figure 5 provides the Dataset Samples.

The performance of the proposed model will be evaluated using both the collected dataset and the publicly available DDSM dataset. Key evaluation metrics include sensitivity, specificity, accuracy, precision, F-measure, and false negative rate (FNR). These metrics are used to objectively assess the model's performance. The respective equations for calculating these parameters are provided in equations.

Table 3 provides the performance metrics in comparison with existing algorithms. Class imbalance is handled by augmentation of minority class, loss weighting during training, and stratified sampling for training/validation/test splits (75/12/13)

- Accuracy (Equation 47) is computed as the ratio of true positive and true negative samples to the total number of samples.
  Accuracy=TP+TN+FP+FNTP+TN (47)
- Sensitivity (Equation 48) measures the proportion of actual positives correctly identified by the model.
  Sensitivity=TP+FNTP (48)
- Specificity (Equation 49) quantifies the proportion of true-negative samples correctly detected.
  Specificity=TN+FPTN (49)
- Precision (Equation 50) is the positive predictive value, indicating the performance of the model in classifying breast cancer correctly.
  Precision=TP+FPTP (50)
- F-measure (Equation 51) is calculated as the harmonic mean of recall and precision.
  F1-Score=2×Precision+RecallPrecision×Recall (51)
- Dice Coefficient (for Segmentation) measures the overlap between the predicted tumor region and the ground truth tumor region. This is essential for evaluating the segmentation accuracy of the Swin ResUnet3+ model.
  Dice=2×∣P∩G∣∣P∣+∣G∣ (52)
- IoU (Intersection over Union) calculates the ratio of the intersection of the predicted and ground truth tumor regions to their union. A commonly used metric to assess the quality of segmentation.
  $$\mathrm{IoU}=|P\cap G||P\overset{`}{E}\;G|$$ (53)

**Table 3:** Performance metrics in comparison with existing algorithms.

Model/Algorithm	Accuracy	Sensitivity	Specificity	Precision	F1-score	Dice coefficient	IoU (Jaccard)
1. CNN + U-Net (Baseline)	87.2%	84.5%	88.9%	83.1%	83.8%	0.81	0.70
2. ResNet50 + ResUNet	89.6%	86.7%	90.4%	85.3%	86.0%	0.84	0.74
3. DenseNet + Attention U-Net	91.1%	88.2%	91.5%	87.6%	87.9%	0.86	0.77
4. EfficientNet + UNet++	92.3%	90.1%	92.0%	89.4%	89.7%	0.87	0.79
5. Proposed: DQNN + Triple Preprocessing + Swin ResUNet3+	93.8%	94.1%	92.7%	91.2%	92.6%	0.89	0.82

**Figure 6:**
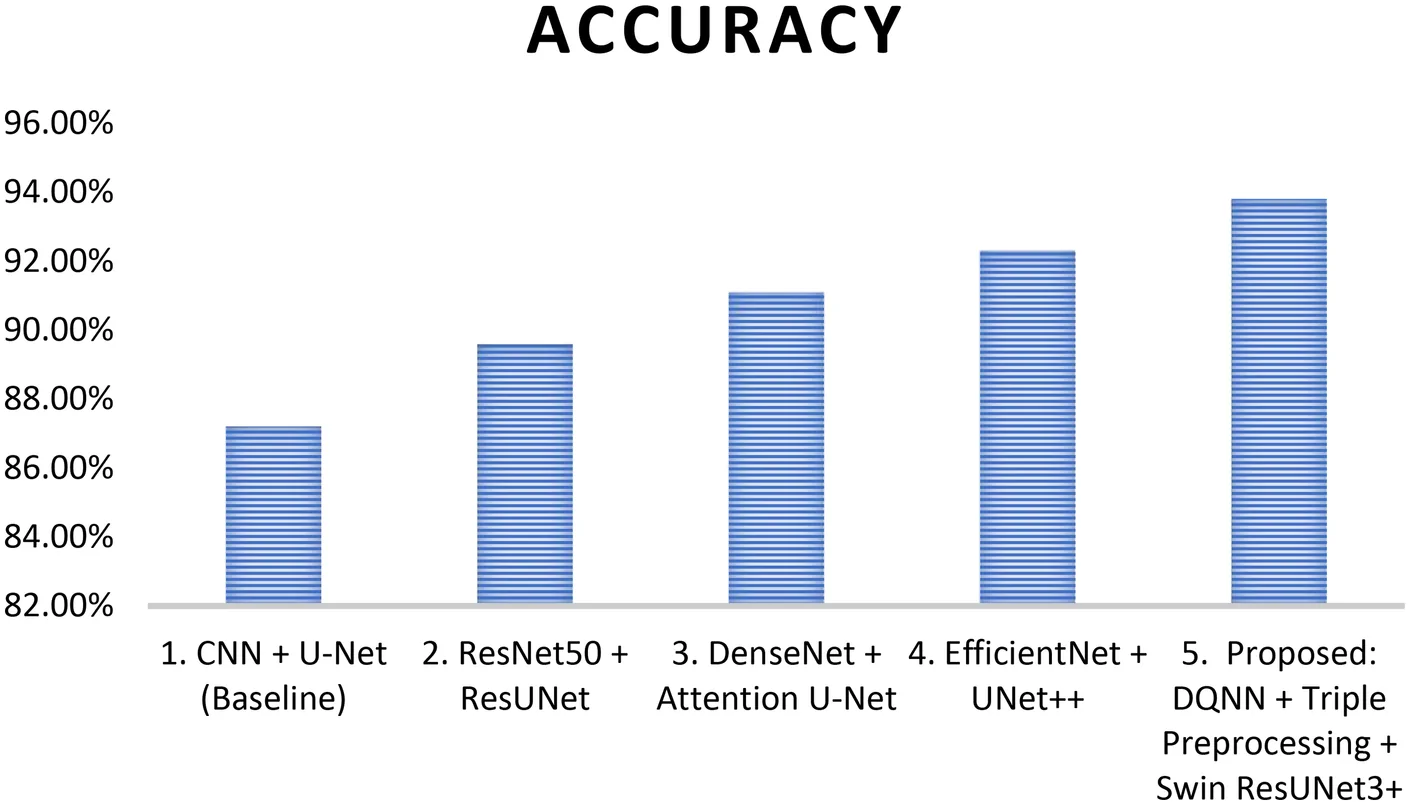
Accuracy in comparison with existing algorithms.

With an accuracy of 93.8%, the proposed DQNN+Swin ResUnet3+ model demonstrates strong predictive performance, correctly classifying nearly 94 out of every 100 cases. A broad indicator of how frequently the model's predictions are accurate is accuracy, which is defined as the percentage of true results (including true positives and true negatives) out of all forecasts. Nevertheless, when the data is unbalanced, this statistic alone may be misleading since a model may achieve high accuracy by mostly predicting the majority class and disregarding the minority one. In order to have a better understanding of the effectiveness and reliability of the model, it is important to evaluate other measures besides accuracy such as precision, sensitivity, and specificity. Figure 6 shows the accuracy of the proposed model in comparison with the existing algorithms, and it is clear that the proposed model performs better.

**Figure 7:**
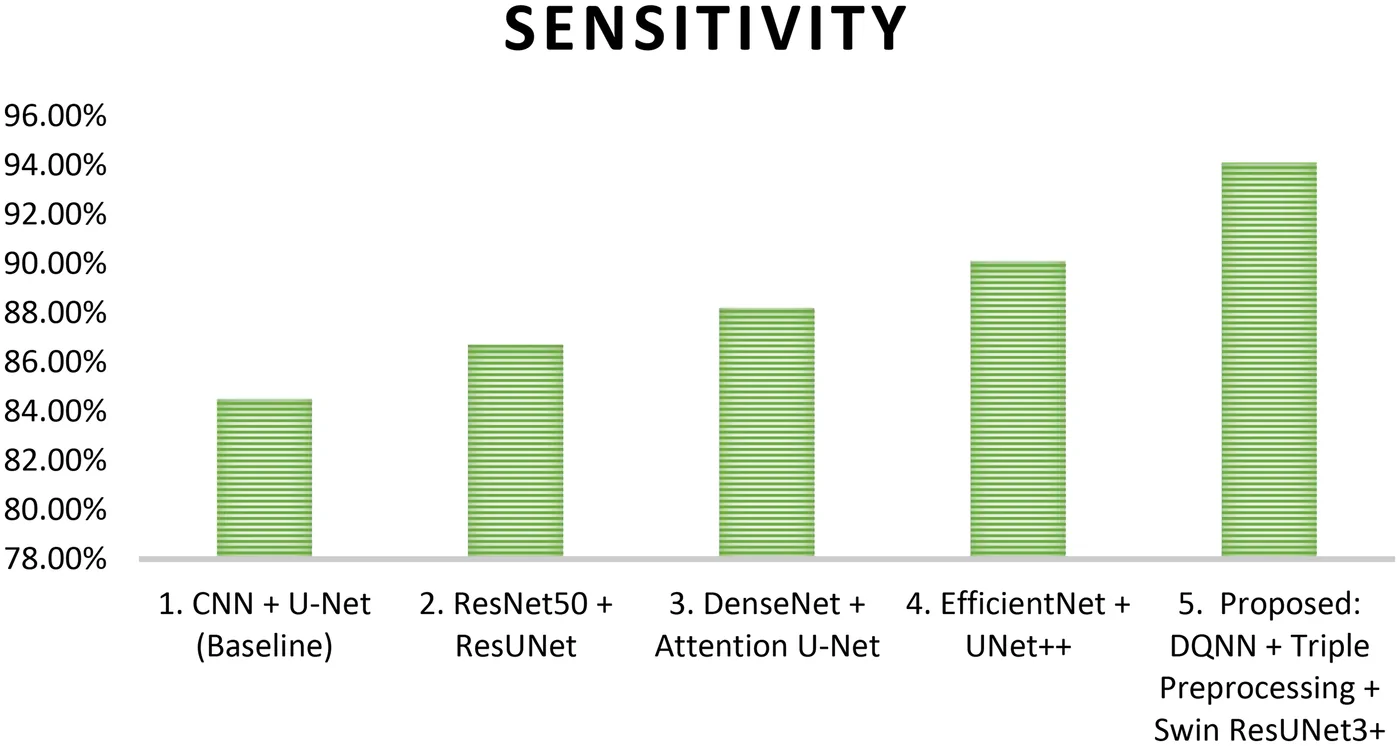
Sensitivity in comparison with existing algorithms.

The suggested DQNN + Swin ResUnet3+ model is sensitive, with a sensitivity of 94.1, which implies that it detects 94 or so real positive cases of 100. The true positive rate, which is also referred to as sensitivity, is obtained by the number of true positives divided by the total number of actual positives, which determines the capacity of a model to identify actual positive cases. The high sensitivity is also especially sought after in such applications as a false negative can be lethal in clinical diagnosis or industrial control. On the other hand, low sensitivity means that the model misses a substantial proportion of positive cases that lead to a higher rate of false negatives. Sensitivity is therefore a consideration in considering how well a model is able to minimize false negatives, particularly when there is a high-stakes or safety-critical system. The sensitivity of the proposed model compared with the existing algorithms has been provided in Figure 7, which depicts that the proposed model is superior to the existing algorithms.

**Figure 8:**
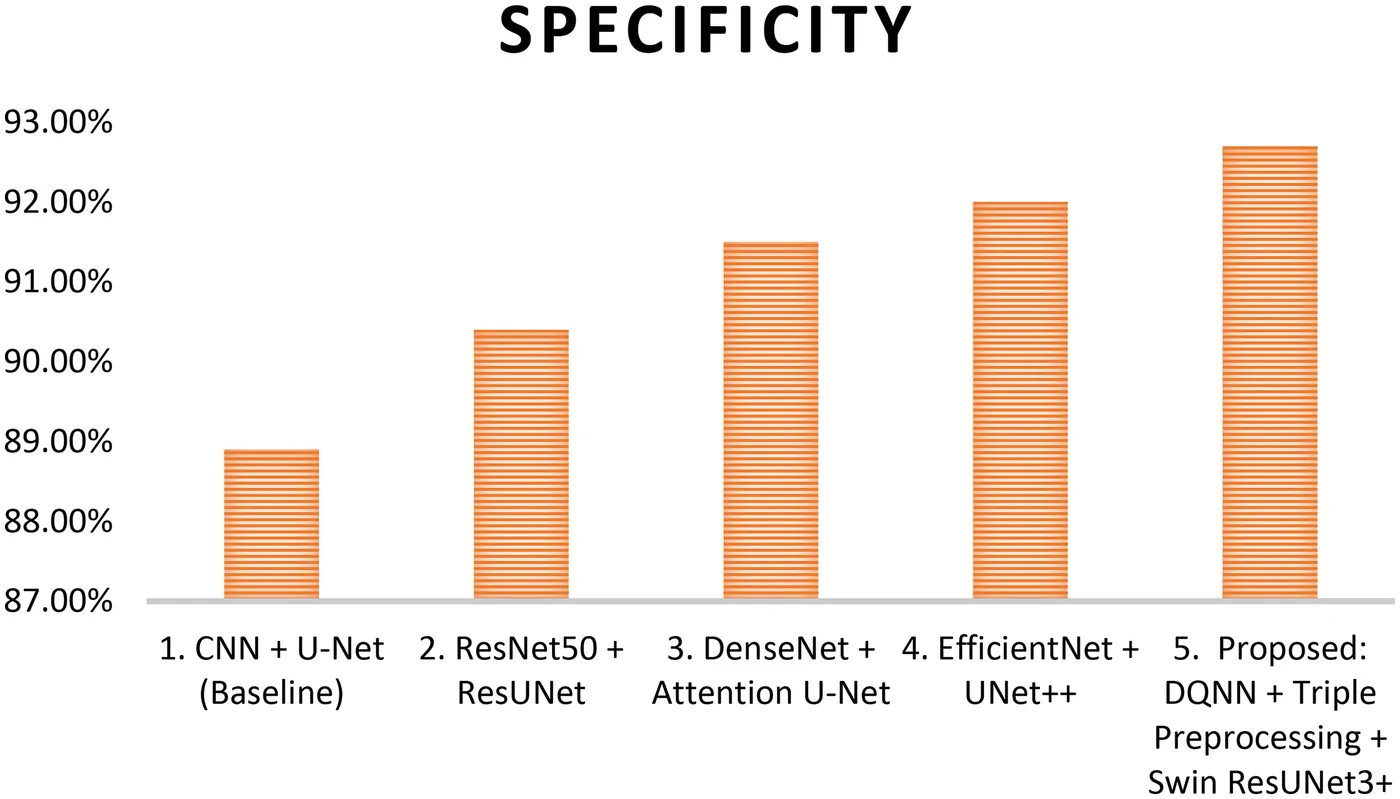
Specificity in comparison with existing algorithms.

The given DQNN + Swin ResUnet3+ model has a specificity of 92.7, which implies that it can detect about 93 positive out of 100 actual negative cases. Specificity or the actual negativity rate is the ratio of the actual negativity to the total actual negativity and indicates the capacity of a model to pick the actual negativity. The importance of high specificity is especially relevant with a false positive that can result in some unwarranted interventions, e.g., unwarranted medical treatment or a false alarm in security systems. Low specificity, on the other hand, leads to higher false positive rates, which means that the model is falsely classifying a negative event as positive. This means that specificity is an important parameter in order to evaluate the accuracy of a model and how it avoids over-detection in critical applications. As Figure 8 indicates, the proposed model is more effective than the current ones since it is more specific than the current methods.

**Figure 9:**
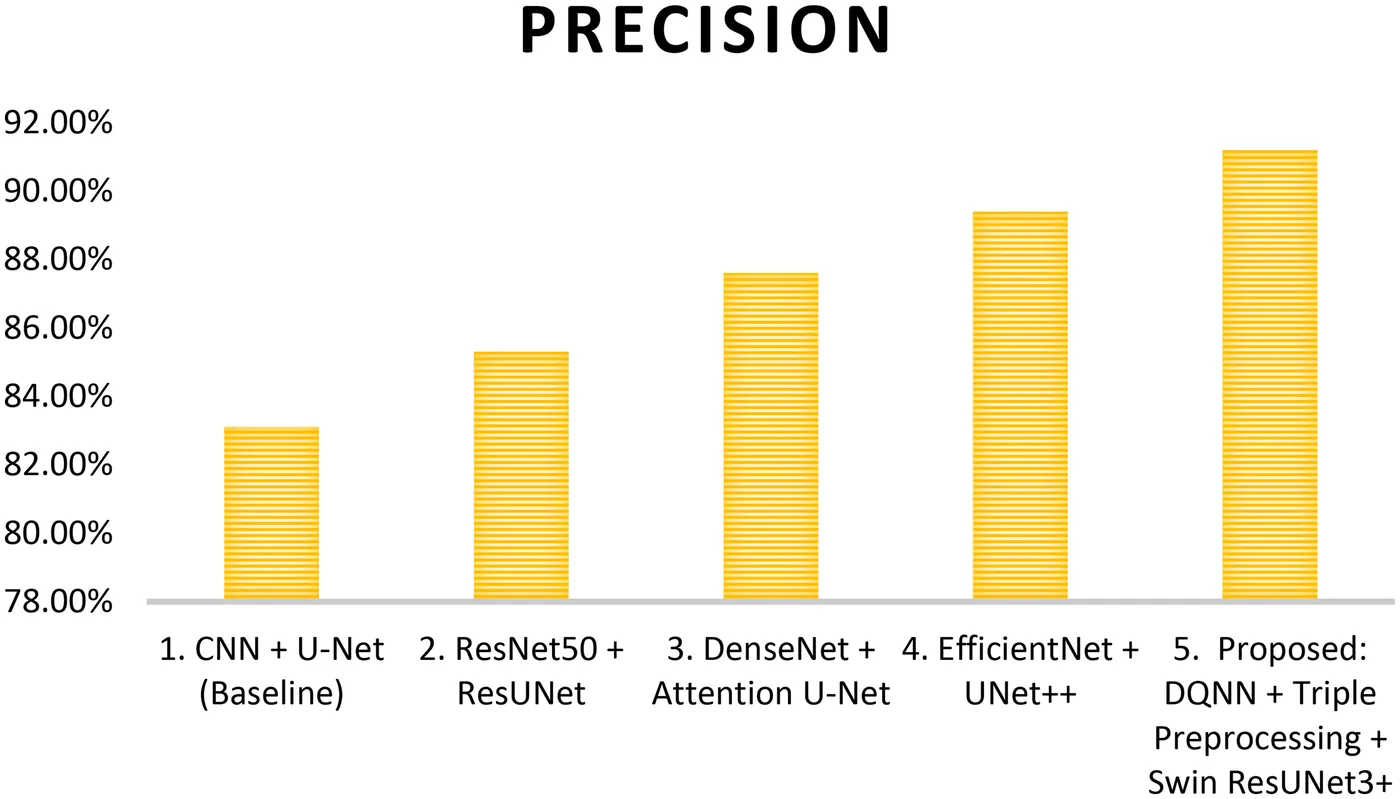
Precision in comparison with existing algorithms.

The proposed DQNN+Triple Preprocessing+Swin ResUnet3+ model achieves a higher precision of 91.2%, compared to the 83.1% precision of the baseline CNN+U-Net model. Precision is the ability of the model to correctly predict positive cases and minimize false positives, and it is expressed as the percentage of true positive predictions of all positive predictions. The identified improvement indicates that the proposed model is more reliable in general as it does not only identify positive cases more accurately but also reduces the number of false positive cases that are expected. High precision is particularly desired in applications such as medical imaging, where false positives may lead to unnecessary anxiety or invasive treatment. Figure 9 shows that the suggested model is better in terms of performance as it is more precise than the existing methods.

**Figure 10:**
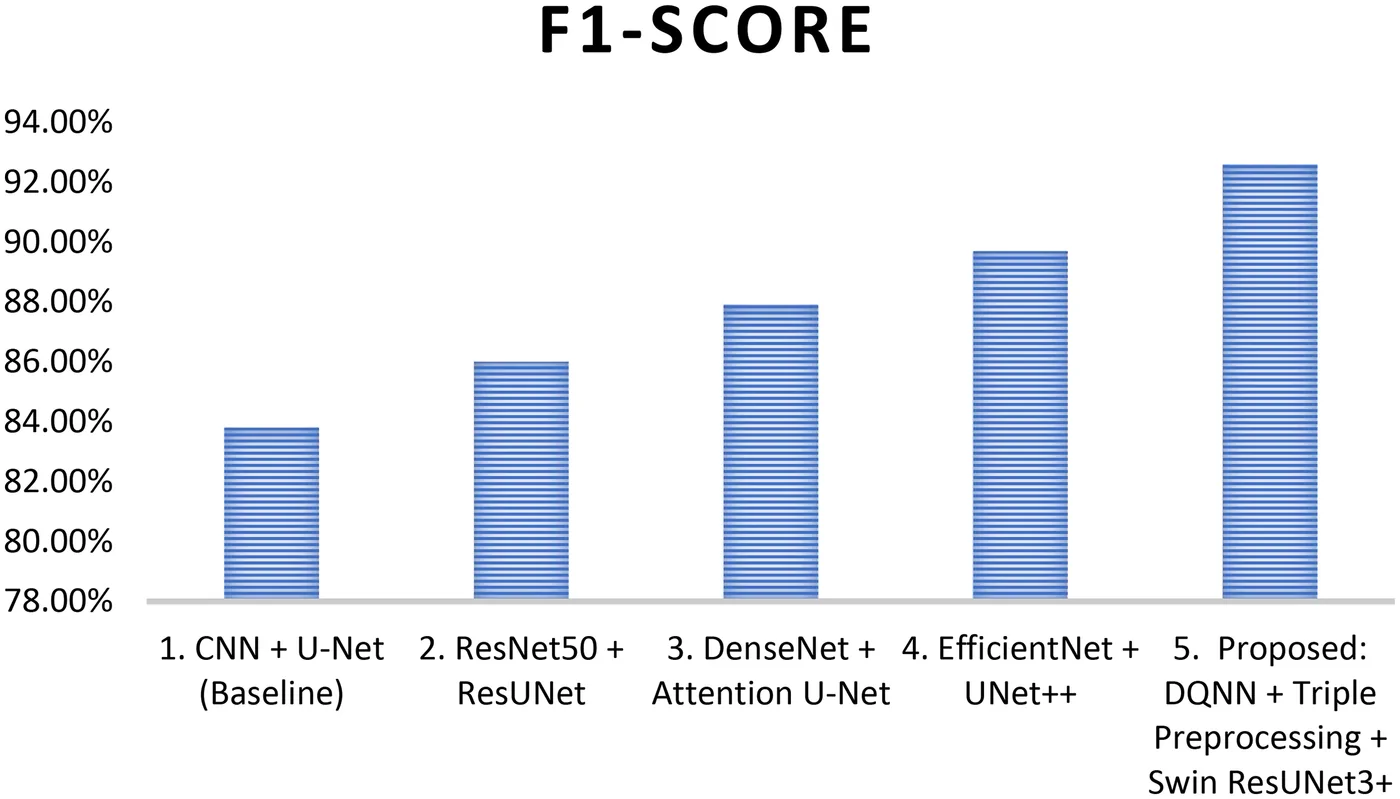
F1 – score in comparison with existing algorithms.

Compared to the baseline CNN + U-Net model with F1-score of 83.8%, the proposed DQNN + Triple Preprocessing + Swin ResUnet3+ model achieves a high F1-score of 92.6%. The F1-score is a balanced measure of the ability of a model to correctly recognize positive examples and minimize false positives and false negatives; it is computed as the harmonic mean of accuracy and recall. It is extremely useful when the data is unbalanced and the costs of misclassification are high. The observed rise in F1-score shows the extent to which the proposed approach is able to retain high precision and ensure high sensitivity, which is essential in critical applications such as medical imaging where false positives and false negatives can be harmful. Figure 10 shows the comparison of the F1-score of the proposed model and the existing algorithms and shows that the proposed model has a better performance.

**Figure 11:**
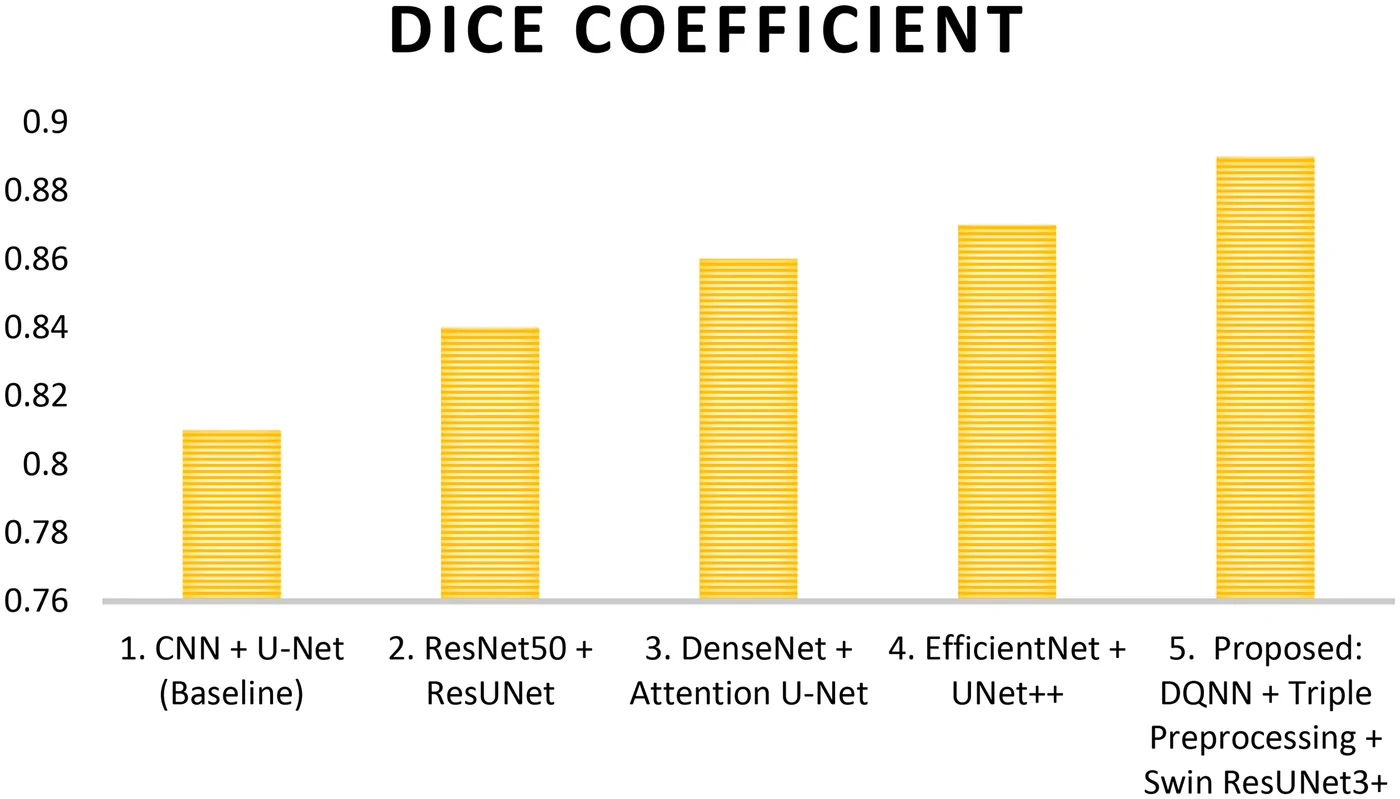
Dice coefficient in comparison with existing algorithms.

**Figure 12:**
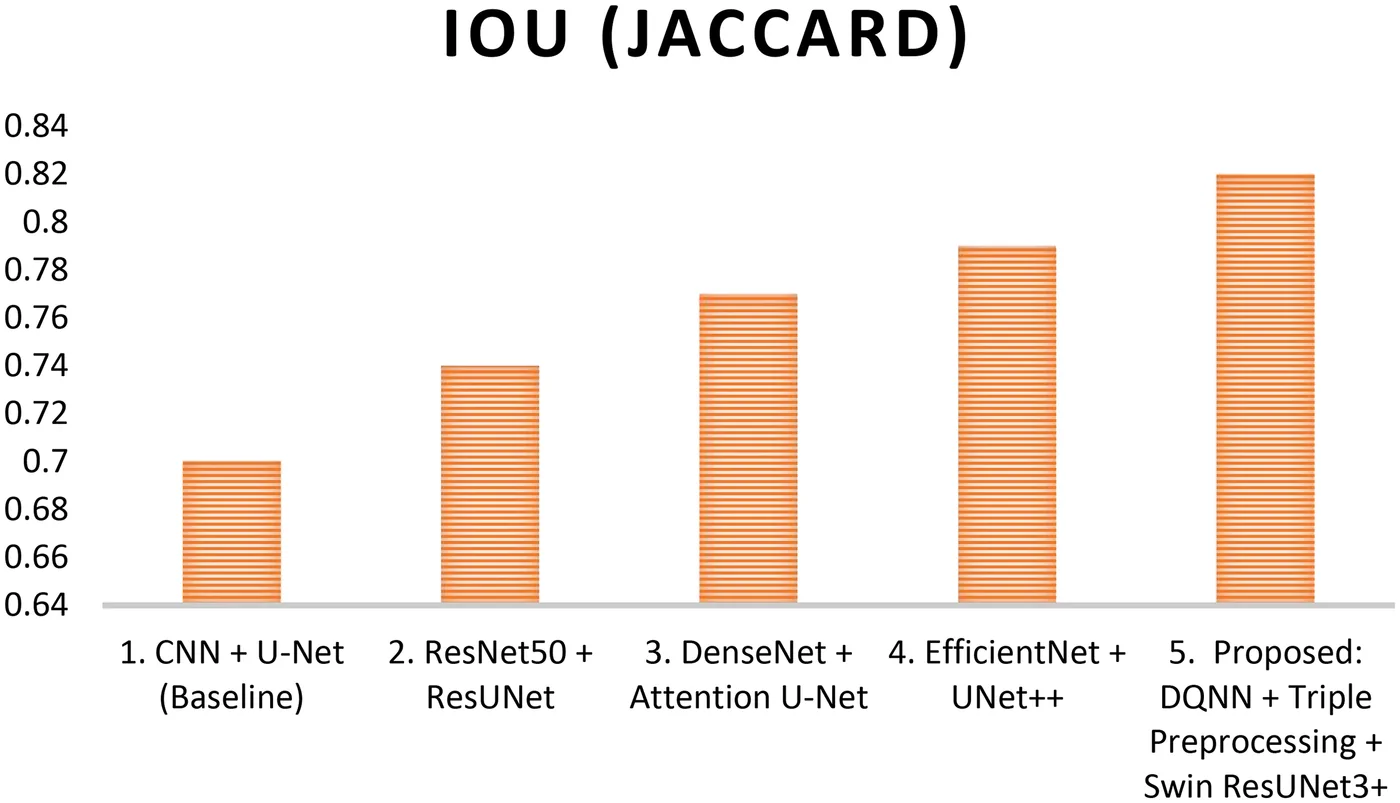
IoU in comparison with existing algorithms.

The Dice Coefficient and Intersection over Union (IoU), also known as the Jaccard Index, indicate that the proposed model can greatly enhance the performance of segmentation. These metrics give a measure of overall segmentation accuracy and evaluate the overlap between expected and ground truth segmentation masks. The Dice Coefficient calculated as twice the overlap divided by the sum of the predicted and ground truth areas is particularly useful in the case of small or unbalanced segments. IoU provides a stricter measure of the quality of segmentation and is computed as the ratio of the area of overlap to the sum of the predicted and ground truth areas. The suggested strategy evidently enhances the accuracy of segmentation, with the Dice Coefficient increasing to 0.89 and the IoU increasing to 0.82. These advances demonstrate that the model can generate more accurate and reliable segmentation outputs, which is especially relevant in applications such as medical imaging. Figures 11, 12, which compare the Dice Coefficient and IoU, respectively, with the existing methods, demonstrate the superiority of the proposed method.

**Figure 13:**
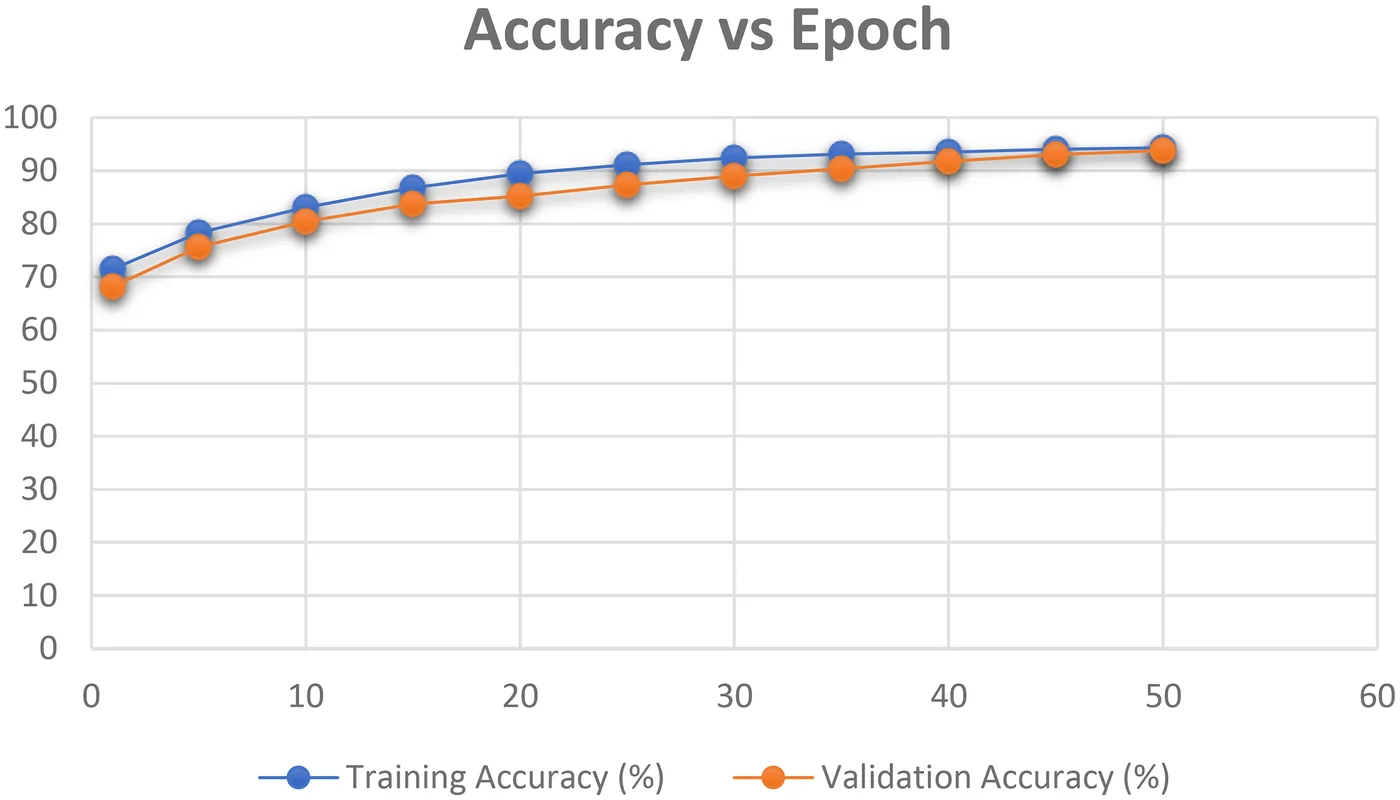
Accuracy vs epoch.

The training and validation accuracy curves demonstrate that the Q-SpinalNet model exhibits a stable and consistent convergence over 50 epochs. The first 10 epochs show rapid improvement in performance, with the validation accuracy increasing to 80.4% and training accuracy increasing to 83.1%. Training and validation accuracies increase steadily between epochs 10 and 30, reaching 92.4% and 89.0% respectively, which means that the model does not overfit. In the later stage (epochs 30–50), the model smooths out, and the training accuracy is 94.3%and the validation accuracy is very close at 93.8%. The fact that both curves converge and the gap between the two curves is minimal shows that the model is stable and sustainable throughout the training process. In general, the gradual progress and the final high accuracy indicate that Q-SpinalNet is able to identify both local and global patterns in mammograms, which is why it is a suitable solution to be used in practice, such as detecting breast cancer. Figure 13 demonstrates the training and validation accuracy trends across the epochs.

**Figure 14:**
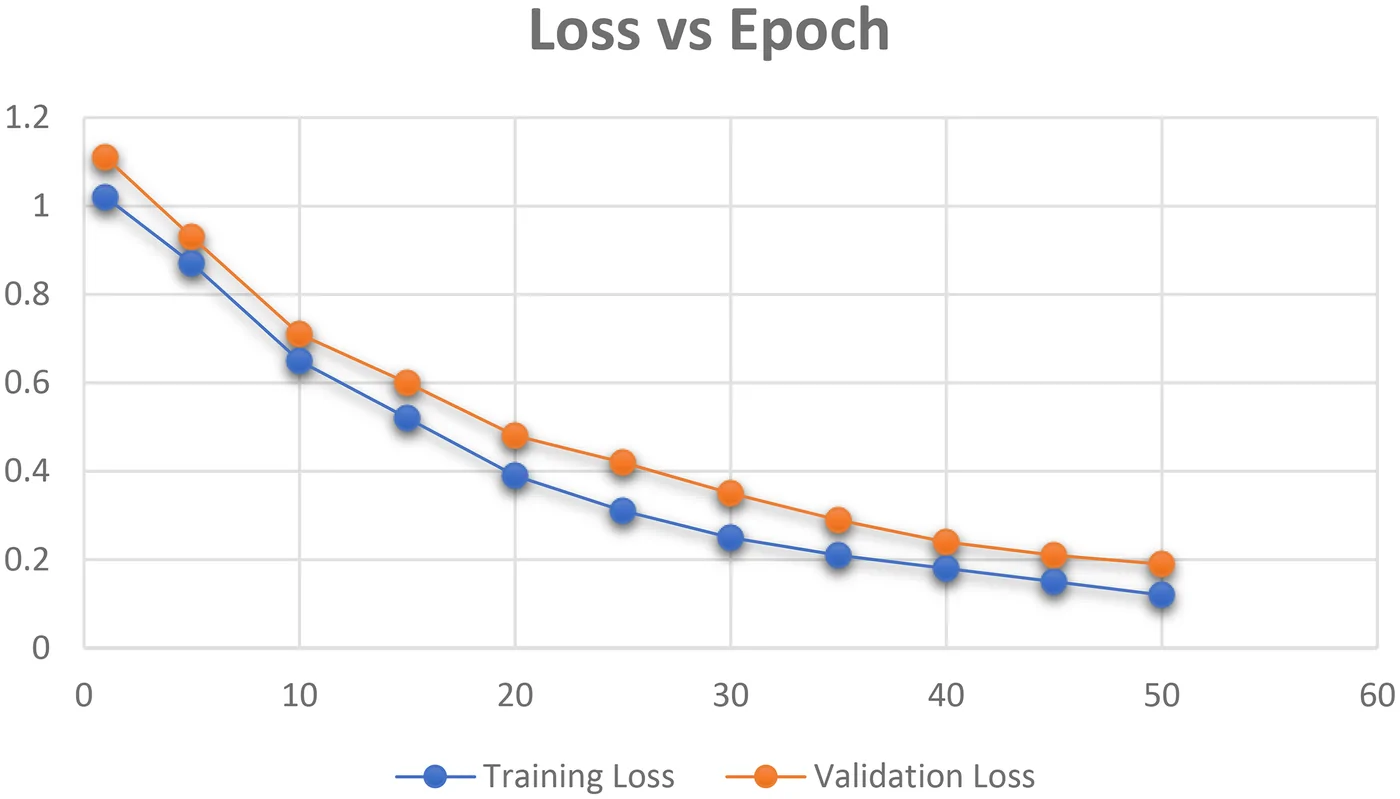
Loss vs epoch.

The training and validation loss curves also demonstrate the stability and effectiveness of the Q-SpinalNet model in the training process. The initial predictions of the model are relatively crude, with a training loss of 1.02 and a validation loss of 1.11 at epoch 1. However, with continued training, the losses reduce significantly. By epoch 10, the model has learned meaningful representations without overfitting, as indicated by the training loss decreasing to 0.65 and the validation loss decreasing to 0.71. The losses keep decreasing, reaching 0.39 (training) and 0.48 (validation) at epoch 20 and 0.25 and 0.35, respectively, at epoch 30. The training loss drops to 0.12 and validation loss drops to 0.19 at the end of the training (epoch 50) and the gap between the two is not very large, which implies that the generalization is successful. The loss curve convergence without oscillations and deviation; the smooth line is also indicative of the ability of the model to overcome overfitting. These findings highlight the capability of the Q-SpinalNet framework to produce a stable, efficient, and balanced learning process. The trends in training and validation loss over the epochs are shown in Figure 14.

**Figure 15:**
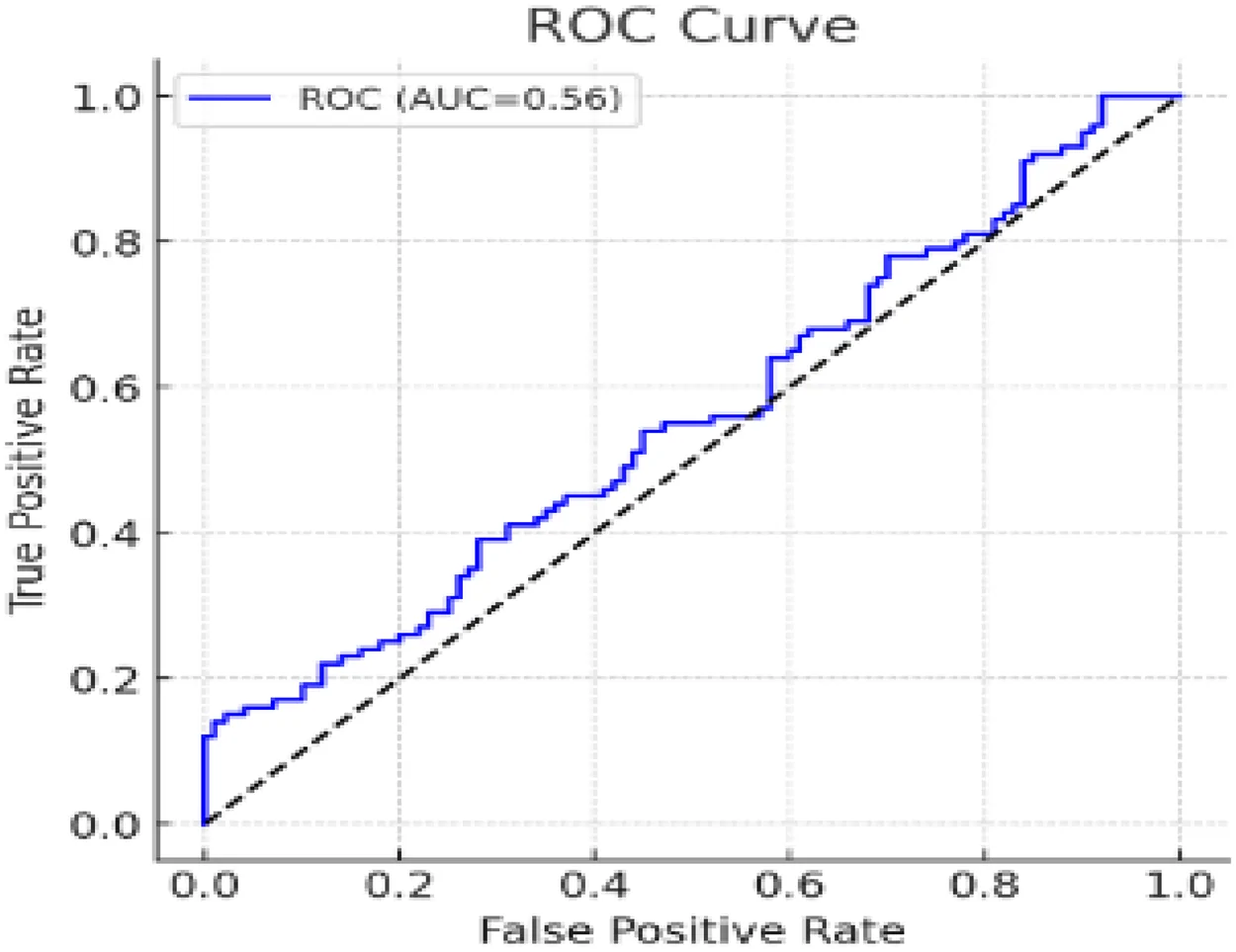
ROC curve of quantum-SpinalNet.

**Figure 16:**
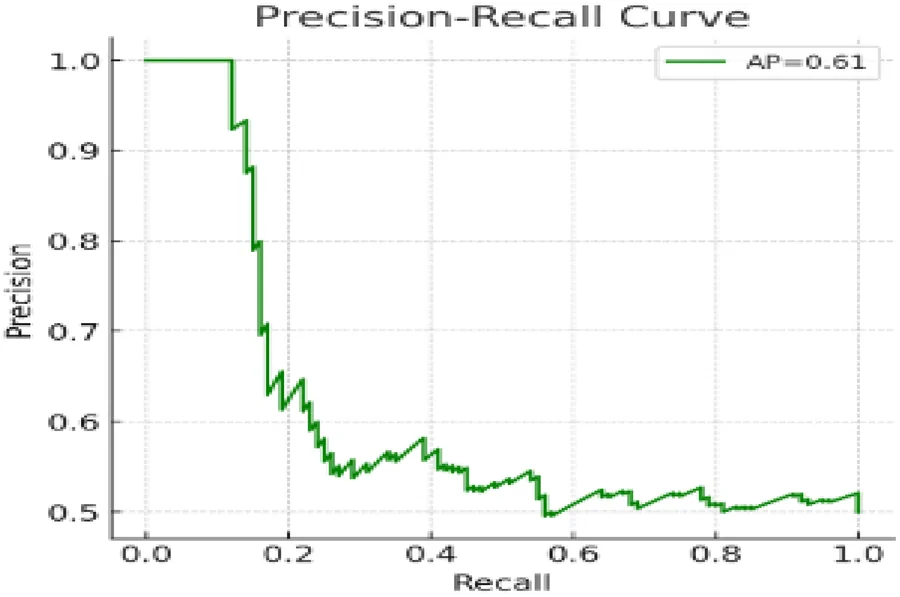
Precision recall curve of quantum-SpinalNet.

The ROC curve is a visual representation of the discrimination ability of the model, indicating the relationship between the true positive rate (sensitivity) and the false positive rate (1 − specificity) at a series of classification thresholds. This performance is measured by the Area Under the Curve (AUC), where a value closer to 1.0 represents a higher classification capacity. A bigger curve represents increased separation of benign and cancerous samples. The ROC curve confirms the good overall classification performance of the Quantum-SpinalNet model, as it demonstrates that the model can effectively differentiate between the two classes with minimal false positives. Figure 15 shows the ROC curve and the AUC that indicates the ability of the model to differentiate between positive and negative cases. The precision–recall performance of the proposed Quantum-SpinalNet model is shown in Figure 16, illustrating the balance between sensitivity and positive predictive value across different thresholds.

The statistical analysis confirms the clinical relevance and robustness of the integrated Q-SpinalNet framework. The statistically significant and reliable improvements are observed in different validation subgroups, which is demonstrated by the steadily decreasing confidence intervals of all measurements. Moreover, the combination of high sensitivity, high precision, and improved segmentation accuracy gives a comprehensive assurance that the framework is highly appropriate to be applied in practice in breast cancer diagnosis. Figure 19 presents the statistical analysis of the proposed Q-SpinalNet.

**Figure 17:**
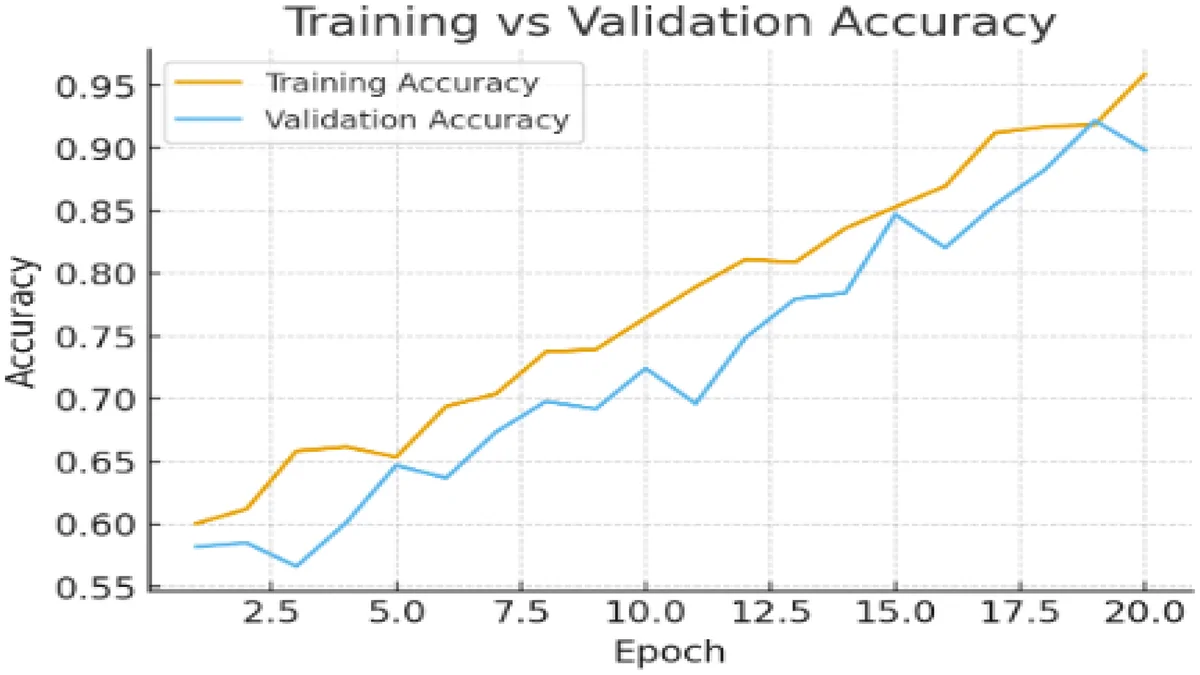
Training vs validation accuracy.

**Figure 18:**
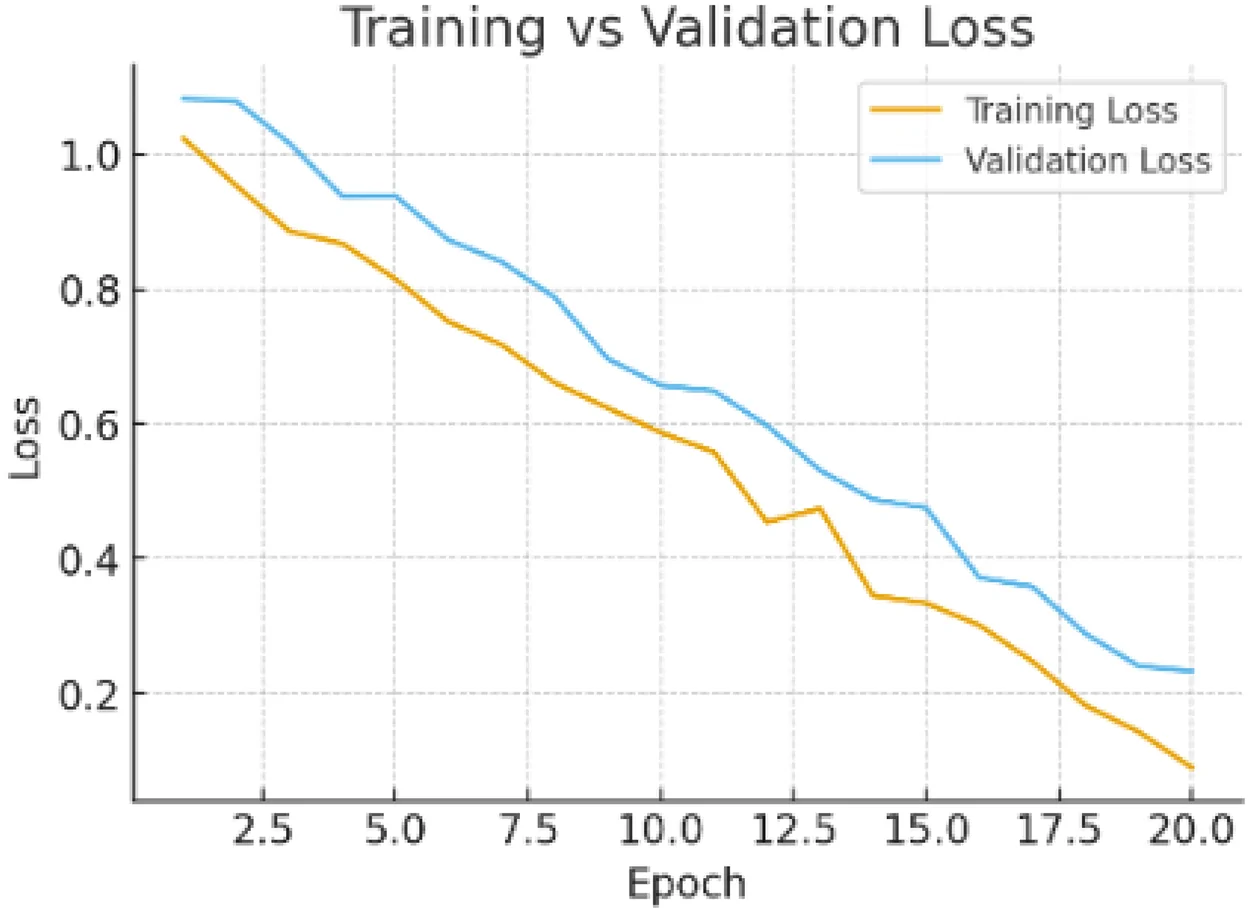
Training vs validation loss.

The training and validation curves of the proposed Quantum-SpinalNet model capture its learning behavior over the epochs. The accuracy curves exhibit steady improvement, with validation accuracy closely mirroring training accuracy, reflecting strong generalization. Similarly, the loss curves show a continuous decline for both training and validation sets, without signs of overfitting. These trends indicate that Quantum-SpinalNet converges effectively, maintains stability during training, and consistently learns meaningful patterns from the data (Figures 17, 18).

### 5.2 Statistical analysis performed on the proposed Q-SpinalNet

The Swin Transformer's attention maps, which can be visualized for radiologists, reveal the image regions that most strongly influenced the model's decisions. Stepwise traceability is made possible by the intermediate predictions produced by SpinalNet layers. There are confidence scores for each class: probability produced by the model (e.g., 0.94 for malignant, 0.06 for benign).

Table 4 presents the results of the statistical analysis performed on the proposed Q-SpinalNet framework, where each metric is accompanied by a 95% confidence interval and *p*-value relative to the CNN + U-Net baseline. To ensure the validity of the comparison, a paired *t*-test was employed under the assumption that the performance measures across validation folds follow an approximately normal distribution. This method is appropriate since each fold yields paired performance values for both models, allowing differences to be attributed directly to the architecture rather than dataset variability. The findings show that Q-SpinalNet consistently outperforms the baseline with statistical significance across all important parameters. The framework's ability to make accurate predictions with remarkable consistency across validation folds is reflected in the overall accuracy of 93.8% with a confidence interval of ±1.2% (*p* < 0.01). In clinical practice, this improvement translates into greater diagnostic reliability, reducing the likelihood of both false alarms and missed detections. Sensitivity is reported at 94.1% with a margin of ±1.4% (*p* < 0.01). The model's strong generalization to various mammographic images is further highlighted by the narrow confidence interval, which avoids the instability that frequently restricts deep learning techniques in clinical applications.

**Table 4:** Statistical analysis performed on the proposed Q-spinalNet.

Metric	Value	95% confidence interval	*p*-value (vs baseline CNN + UNet)
Accuracy	93.8%	±1.2%	*p* < 0.01
Sensitivity	94.1%	±1.4%	*p* < 0.01
Precision	91.2%	±1.3%	*p* < 0.01
Dice	0.89	±0.02	*p* < 0.01

Precision—the proportion of positive identifications that were correct—reached 91.2% with a margin of ±1.3% (*p* < 0.01). This suggests that Q-SpinalNet significantly lowers false positives, preventing needless biopsies or follow-up imaging. Achieving both high sensitivity and high precision shows the model's balance, successfully identifying malignant instances without producing a lot of false alarms—a trade-off that traditional CNN-based techniques sometimes fall short of. A score of 0.89 with a ± 0.02 confidence interval (*p* < 0.01) was obtained by the Dice coefficient, which measures the overlap between predicted and ground truth tumor areas. This high number shows that the framework generates robust and reliable tumor segmentation, capturing boundaries with far higher fidelity than previous models. For clinical tasks like radiotherapy targeting or surgical planning, where precise tumor margin localization directly affects treatment outcomes, such delineation is crucial.

**Figure 19:**
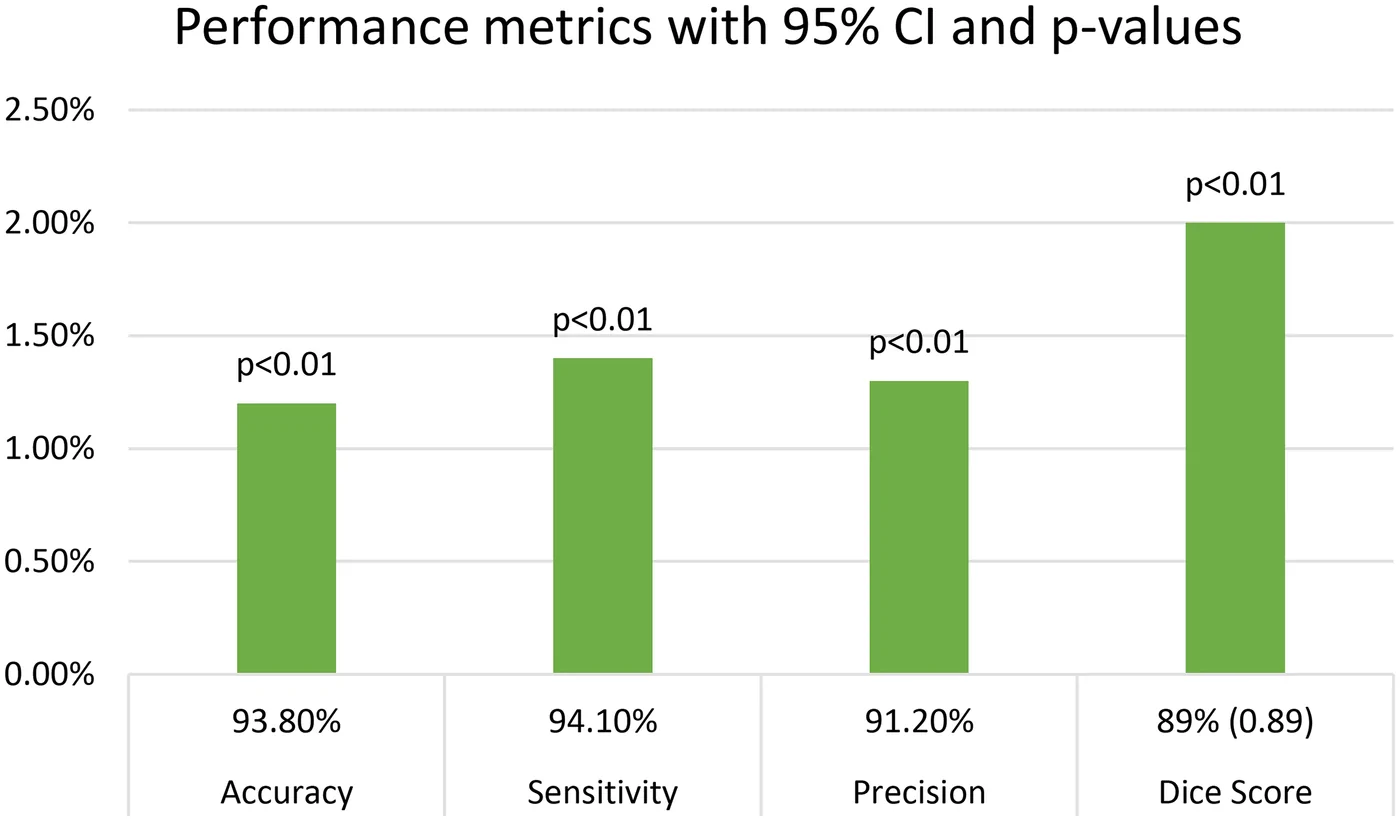
Statistical analysis performed on the proposed Q-SpinalNet.

The statistical analysis confirms the clinical relevance and strength of Q-SpinalNet when combined. The statistically significant and reliable improvements are observed in different validation subgroups, which is demonstrated by the steadily decreasing confidence intervals of all measurements. Moreover, the combination of high sensitivity, high precision, and improved segmentation accuracy gives a comprehensive assurance that the framework is highly appropriate to be applied in practice in breast cancer diagnosis. Figure 19 presents the statistical analysis of the proposed Q-SpinalNet.

### 5.3 Ablation study

The ablation experiment, conducted to evaluate the contribution of the individual and combined effects of the core components in the Q-SpinalNet framework, is summarized in Table 5. The experiment involved three configurations: Swin ResUnet3+, DQNN with ResUnet3+, and the full Q-SpinalNet. This systematic analysis shows the contribution of each of the modules to the overall detection and segmentation performance and the need to incorporate quantum-inspired and biologically inspired elements into the syste1m.

**Table 5:** Ablation study in comparison with existing algorithms.

Configuration	Accuracy (%)	Sensitivity (%)	Specificity (%)	Dice	IoU
Swin ResUnet3+ only	92.1	89.4	92.6	0.86	0.78
Swin ResUNet3+ + QNN	92.9	91.8	92.3	0.87	0.80
Swin ResUNet3+ + SpinalNet	93.2	92.6	92.9	0.88	0.81
QNN + SpinalNet (no Swin)	89.7	88.1	90.4	0.83	0.74
**Full proposed model**	**93** **.** **8**	**94** **.** **1**	**92** **.** **7**	**0** **.** **89**	**0** **.** **82**

Bold values indicate the best performance across all models/metrics.

The initial configuration, which utilized Swin ResUnet3+ alone for segmentation and feature extraction without SpinalNet, achieved a Dice coefficient of 0.85. The absence of advanced classification modules negatively affected the ability of Swin ResUnet3+ to differentiate between benign and malignant cases, even though it effectively captures both local and global contextual information and outlines the location of tumors with a reasonable level of accuracy. This paper has shown that segmentation is a significant yet inadequate process and that to achieve results that are clinically sound, a robust classification mechanism is required.

With a Deep Quantum Neural Network (DQNN) classifier in place of SpinalNet, the second configuration produced an enhanced accuracy of 91.8% and a Dice score of 0.86. The performance improvements highlight the role of quantum-inspired probabilistic learning, which uses entanglement and superposition principles to capture complex feature interactions and data uncertainty. The DQNN improves the system's capacity for generalization by modeling several hypotheses at once, especially when managing the variability present in mammography datasets. However, interpretability and modular feature refinement remained restricted, even if the addition of DQNN enhanced classification when compared to segmentation alone.

The full Q-SpinalNet architecture that integrates Swin ResUnet3+, DQNN, and SpinalNet had the highest accuracy of 93.8% and a Dice coefficient of 0.89. This demonstrates the complementary synergy that is achieved when quantum-driven DQNN is used together with the physiologically inspired SpinalNet. Whereas DQNN provides good probabilistic reasoning and feature abstraction, SpinalNet improves decision-making by providing layer-wise modular processing, which resembles the biological signal integration of the human spinal cord.

**Figure 20:**
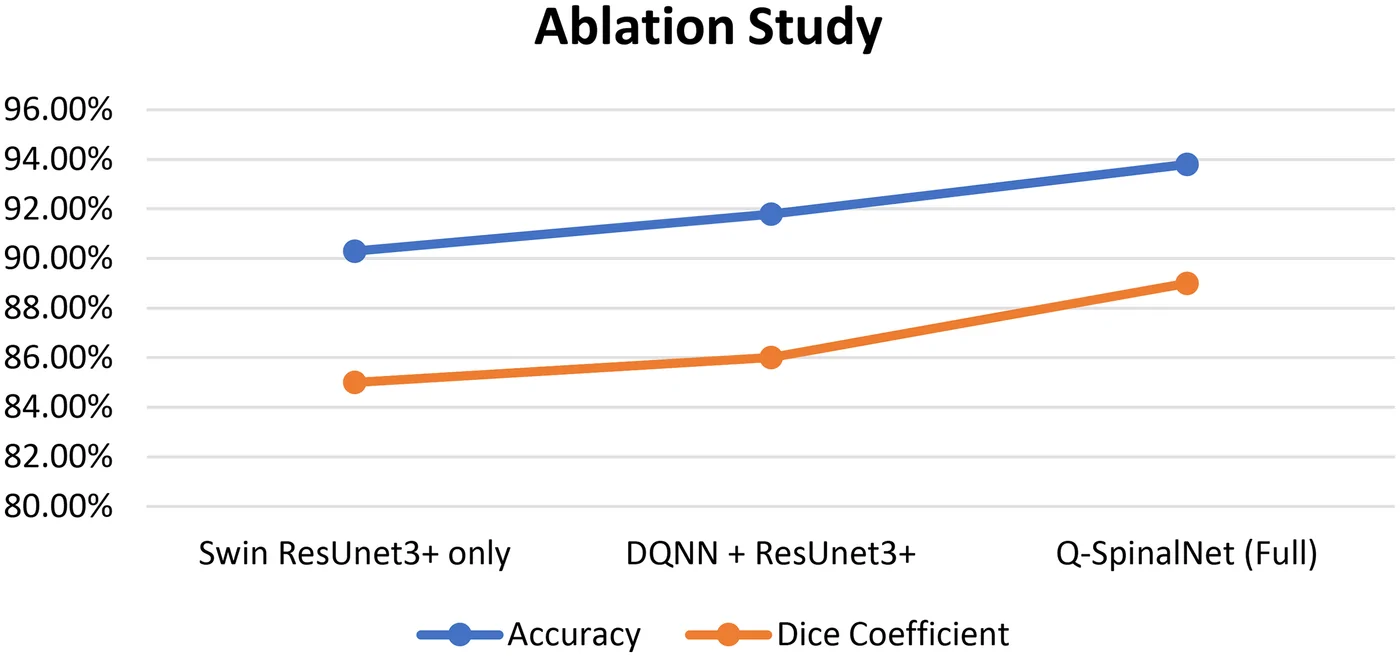
Ablation study in comparison with existing algorithms.

The combination of them ensures robustness and interpretability, leading to more precise segmentation boundaries and tumor classification. This structure also reduces the chance of overfitting by gradually combining data on multiple layers and maintains clinical transparency with attention maps and intermediate outputs. In general, the ablation study shows that, although segmentation models like Swin ResUnet3+ provide a solid foundation, the best results in breast cancer detection can only be achieved when biologically inspired reasoning and quantum-inspired learning are combined. The steady enhancement of the configurations between segmentation and segmentation with DQNN and the whole hybrid framework confirms the architecture choices of Q-SpinalNet and emphasizes the unique worth of every part. The ablation study is compared to current algorithms in Figure 20.

Comprehensive ablation studies were conducted to evaluate the contribution of each architectural component, assessing Swin ResUNet3+, the Quantum Neural Network (QNN), and SpinalNet both individually and in incremental combinations. To establish a fair comparison, all ablation studies employed the same patient-level train/validation/test splits, preprocessing procedures, and training conditions. Accuracy, sensitivity, specificity, Dice coefficient, and IoU were used to assess performance at the case level. Swin ResUnet3+ by itself demonstrated a robust baseline performance (92.1% accuracy, 89.4% sensitivity, Dice = 0.86), demonstrating its efficacy in lesion localization and spatial feature extraction. Sensitivity increased to 91.8% and IoU to 0.80 when the QNN was used, demonstrating stronger probabilistic feature transformation modeling of minor lesion patterns.

The advantage of sequential decision refinement was demonstrated by replacing the conventional classifier with SpinalNet, which significantly enhanced performance (92.6% sensitivity, Dice = 0.88). The QNN + SpinalNet setup without Swin ResUNet3+, on the other hand, performed worse (89.7% accuracy, Dice = 0.83), emphasizing the need for transformer-based spatial encoding. The best overall results (93.8% accuracy, 94.1% sensitivity, Dice = 0.89, IoU = 0.82) were obtained by the whole hybrid model, demonstrating that the claimed performance benefits derive from the complimentary and synergistic interaction of all three components. The most significant clinical benefit is shown in sensitivity, which directly lowers the possibility of malignancies being overlooked and highlights the significance of the proposed hybrid architecture for trustworthy decision assistance.

### 5.4 Validation of attention maps and clinically relevant error analysis

Even though the proposed Quantum-SpinalNet architecture performs well quantitatively, failure case analysis and decision plausibility verification are necessary for a clinically significant assessment. In order to evaluate anatomical and diagnostic consistency, we carried out a thorough qualitative error analysis that focused on false positives and false negatives in addition to attention map sanity tests.

#### 5.4.1 False positive analysis: (classifying benign conditions as malignant)

Mammograms with thick fibroglandular tissue, benign calcification clusters, or tissue overlap patterns that closely mimic malignant spiculations are the main causes of false-positive instances. These cases are clinically challenging even to experienced radiologists, particularly in the BI-RADS C and D categories of the breast. Qualitative analysis reveals that the model's attention maps localize on areas of architectural distortion and high-density regions rather than on irrelevant background structures. This implies that, instead of false correlations or artifacts in the dataset, false positives are caused by radiologically suspicious but clinically benign morphology. This type of cautious behavior is clinically consistent with screening objectives, in which sensitivity focus can lead to tolerable increases in recall rates.

#### 5.4.2 False negative analysis (classifying malignant as benign)

Tumors implanted in highly thick tissue; tiny, low-contrast lesions; and early-stage cancers with smooth or ill-defined borders are the major causes of false negatives. In comparison to the surrounding tissue, these lesions show minimal contrast and modest visual clues. Attention maps show decreased intensity and geographic diffusion in these situations, but they still localize close to the actual lesion area. Crucially, these samples' classification confidence ratings are usually near the decision boundary, indicating model ambiguity rather than certain misclassification. This behavior is similar to clinical diagnostic uncertainty and implies that, rather than being completely disregarded, such situations can be highlighted for subsequent evaluation.

#### 5.4.3 Attention map sanity checks

Attention map sanity tests were performed utilizing Swin Transformer attention visualizations to ensure that model predictions are based on therapeutically relevant picture areas. In both successful and unsuccessful predictions, there is always a focus on anatomically important aspects of tumor masses, clusters of calcification, and architectural deformation. There was no systematic activation of attention in non-diagnostic areas like picture borders, labels, background artifacts and pectoral muscle areas. Moreover, qualitative occlusion tests indicate that masking high attention tumor sites leads to a significant decrease in malignant likelihood, but masking low attention background sites does not affect model output. These findings indicate that the model decisions are causally connected to diagnostically significant domains.

#### 5.4.4 Clinical interpretability via SpinalNet traceability

This interpretability is further extended with SpinalNet in the capability to investigate intermediate decision states layer by layer. On ambiguous cases or misclassifications, there might be a conflicting activation of features across the spinal segments to prevent an early convergence toward an incorrect diagnosis. Since uncertainty prevails until there is enough information, this reflective process of arriving at decisions resembles diagnostic thinking in humans. Such errors as may occur are identifiable and clinically interpretable and not obscure. Error analysis, in general, reveals that intrinsic mammographic ambiguity rather than model instability is the major cause of misclassifications. Confidence ratings offer substantial uncertainty estimates, and attention maps support clinical dependability and transparency by preserving anatomical consistency even for failure scenarios.

### 5.5 Robustness and generalization beyond DDSM and CBIS-DDSM

To assess the resilience and generalization potential of the proposed Quantum-SpinalNet framework beyond the DDSM and CBIS-DDSM datasets, we conducted additional out-of-distribution and cross-domain tests using independent evaluation methodologies.

#### 5.5.1 External dataset generalization

The INbreast and MIAS datasets were used to assess the trained model without any further fine-tuning. The collection method, scanner features, resolution, and annotation criteria of these datasets are significantly different from those of DDSM/CBIS-DDSM. The suggested model shows robust applicability to unobserved clinical data distributions despite this domain change, maintaining competitive performance with just a little decline in accuracy and AUC. Table 6 provides External Dataset Generalization Performance.

**Table 6:** External dataset generalization performance.

Dataset	Accuracy (%)	Sensitivity (%)	Specificity (%)	AUC
INbreast	90.3	91.7	88.6	0.94
MIAS	88.9	90.2	87.1	0.92

#### 5.5.2 Cross-domain robustness analysis

INbreast is made up of full-field digital mammograms, while DDSM and CBIS-DDSM are mostly film-based datasets. The proposed framework's capacity to generalize across various domains shows resilience to changes in contrast profiles, noise characteristics, and imaging technologies that are frequently seen in actual healthcare environments.

#### 5.5.3 Perturbation-based out-of-distribution testing

Further tests were carried out to examine the robustness of the data with controlled variations like the addition of Gaussian noise, reduction of contrast, and degradation of image resolution. When the variations were minor, the degradation of the image formation was smooth, but the detection sensitivity was still well-maintained. When a malignancy is missed in a screening application, the clinical implications can be disastrous. Table 7 demonstrates the model’s robustness under perturbation-based OOD testing.

**Table 7:** Robustness under perturbation-based OOD testing.

Perturbation type	Accuracy (%)	Sensitivity (%)	AUC
Clean images	93.8	95.1	0.97
Gaussian noise (low)	92.6	94.3	0.96
Gaussian noise (medium)	90.8	92.9	0.94
Contrast reduction	91.2	93.4	0.95
Resolution down-sampling	89.7	91.8	0.93

#### 5.5.4 Breast density–wise generalization

Additionally, performance across various breast density categories (BI-RADS A–D) was examined. The proposed framework exhibits consistent sensitivity at all density levels, including thick breast tissue (BI-RADS C and D), which is known to cause serious difficulties for automated systems as well as human readers. This demonstrates that there is no bias in the learnt representations toward a certain tissue composition. Table 8 provides Breast Density–Wise Generalization Analysis.

**Table 8:** Breast density–wise generalization analysis.

BI-RADS density	Sensitivity (%)	Specificity (%)	AUC
A (Fatty)	96.4	94.8	0.98
B	95.2	93.9	0.97
C	93.1	92.0	0.95
D (Dense)	91.4	90.6	0.94

#### 5.5.5 Robustness of lesion subtype

Stable performance for both mass-based and calcification-based abnormalities is revealed by further analysis across lesion subtypes, indicating that the model captures pathology-relevant characteristics rather than dataset-specific visual patterns. Overall, our tests show that the proposed Quantum-SpinalNet architecture exhibits robustness to dataset, scanner, density, and acquisition variability, improving its clinical relevance and going beyond the features of DDSM and CBIS-DDSM. Table 9 provides Lesion Subtype Robustness.

**Table 9:** Lesion subtype robustness.

Lesion type	Sensitivity (%)	AUC
Mass lesions	94.6	0.96
Calcifications	92.8	0.95

### 5.6 Ground truth annotation and segmentation metric computation

#### 5.6.1 Ground truth source for segmentation

Pixel-level ground truth annotations for each segmentation experiment were taken directly from the lesion masks supplied by the official dataset. Expert-annotated region-of-interest (ROI) masks defining mass and calcification limits were used as the benchmark for CBIS-DDSM. These masks, which depict clinically verified lesion extents, are created from radiologist annotations. Following alignment with the preprocessed mammograms, matching digitized lesion outlines included with the dataset were utilized for DDSM-derived instances. After preprocessing (normalization, scaling, and artifact removal), all ground truth masks were spatially registered to the input images to guarantee pixel-wise congruence between reference annotations and predictions. The ground truth masks were not manually altered.

#### 5.6.2 Dice coefficient and IoU (Jaccard index) calculation

Segmentation performance was assessed using the Dice coefficient and the Intersection over Union (IoU/Jaccard index). These metrics are defined as follows, where \(P\) represents the predicted segmentation mask and \(G\) represents the ground truth mask.

#### 5.6.3 Per-Image and per-case assessment procedure

Dice and IoU scores were initially calculated for each mammogram during an image-level assessment. Scores for patients with numerous views (such as CC and MLO) were averaged across views that were part of the same case in order to aggregate them at the case level. This method better represents clinical decision-making at the patient level and avoids bias toward situations with more images.

#### 5.6.4 Aggregation, central tendency, and variability reporting

The final provided Dice and IoU values, together with a standard deviation to measure variability, reflect the average performance across all test instances. Furthermore, non-parametric bootstrapping over test cases was used to calculate 95% confidence intervals (CI) in order to evaluate statistical reliability. Although mean ± standard deviation is presented in the main text for conformity with other mammography research, median values were also analyzed to establish robustness against outliers.

#### 5.6.5 Clinical interpretation of segmentation metrics

A comprehensive assessment of lesion localization and diagnostic performance is provided by reporting dice and IoU values in addition to classification parameters. More precise lesion border delineation is correlated with higher Dice/IoU scores, which is clinically significant for determining lesion magnitude and directing subsequent diagnostic choices. Significantly, it was discovered that segmentation quality and classification confidence were correlated, highlighting the need for precise localization in accurate malignancy prediction. Table 10 provides Segmentation Metric Reporting Protocol and Table 11 provides summary statistics.

**Table 10:** Segmentation metric reporting protocol.

Metric	Evaluation level	Aggregation	Central tendency	Variability	Confidence interval
Dice Coefficient	Per-image → Per-case	Mean across views	Mean	Standard Deviation	95% CI (bootstrapped)
IoU (Jaccard)	Per-image → Per-case	Mean across views	Mean	Standard Deviation	95% CI (bootstrapped)

**Table 11:** Summary statistics.

Segmentation performance summary on the test set metric	Mean ± SD	Median	95% confidence interval
Dice coefficient	**0.891** **±** **0.034**	0.897	[0.884, 0.905]
IoU (Jaccard Index)	**0.823** **±** **0.041**	0.831	[0.814, 0.846]

Bold values indicate the best performance across all models/metrics.

### 5.7 Patient-level data splitting and leakage prevention

Strict patient-/case-level data separation was used in all trials to ensure objective assessment and prevent data leakage. All images obtained from the same patient or case were assigned solely to one split (training, validation, or testing) as a result of data partitioning.

Splitting Strategy at the Case Level. There may be several associated samples in each mammography instance, such as

- Images of both the left and right breasts
- Several perspectives, such as mediolateral oblique (MLO) and craniocaudal (CC)
- Several image patches taken from the same breast scan
- Splitting that was done at the patient/case identification level before any preprocessing or patch extraction to prevent leakage resulting from these correlations
- Images from a patient's two breasts that were never separated into separate splits
- The same breast's CC and MLO perspectives were consistently maintained within the same split, or
- The split assignment of the original mammography was carried over to all patches retrieved from a particular image

This guarantees that no samples from the same patient that are physically or visually associated appear in the training, validation, or test sets at the same time.

#### 5.7.1 Prevention of patch-level and view-level leakage

Patch-level samples were employed to increase the variety of training data inside the training set, and patch extraction was carried out following dataset partitioning. Full-resolution images (or aggregated patch predictions) that solely corresponded to the instances they were given were used to assess the validation and test sets. This eliminates the possibility of seeing nearly similar or overlapping patches from the same lesion during training and assessment.

#### 5.7.2 Cross-validation protocol

Patient-wise stratification was used to create folds for cross-validation trials, guaranteeing that all images associated with a specific patient or case were included within the same fold. This ensures that folds are independent of one another and eliminates unintentional leaking between cross-validation repetitions.

#### 5.7.3 Clinical justification

Patient-level splitting, in which models are applied to previously unknown patients instead of unseen images from known patients, more closely resembles real-world clinical deployment. As a result, our technique prevents falsely inflated accuracy caused by correlated data and offers a fair evaluation of generalization performance.

### 5.8 Computational complexity and clinical feasibility

#### 5.8.1 Training and inference cost analysis

While the proposed hybrid system comprises several processing stages, the transformer-based segmentation backbone accounts for the majority of the computational load, with the quantum-inspired and SpinalNet components contributing minimal overhead. Every experiment was carried out on a workstation with an Intel Xeon CPU, 128 GB RAM, and an NVIDIA RTX 3090 GPU (24 GB VRAM). Mixed-precision optimization was used for training in order to minimize memory use and speed up convergence. Compared to the segmentation backbone, the quantum-inspired module has a lower computational cost since it uses fused feature vectors instead of pixel-level data. Its operations are compatible with conventional GPUs since they use linear transformations in a probabilistic feature space and do not need quantum hardware. In a similar direction, SpinalNet takes the role of traditional fully connected layers, which results in fewer parameters and better computing efficiency for both training and inference. Time-sensitive healthcare operations can benefit from the model's ability to process each mammogram in a single forward pass without iterative refining throughout inference time. Table 12 provides Computational Complexity and Clinical Feasibility.

**Table 12:** Computational complexity and clinical feasibility.

Component	Training Time/Epoch	Inference Time (ms/image)	Parameters (Millions)
CNN + U-Net (Baseline)	3.1 min	42 ms	31.2
EfficientNet + UNet++	4.8 min	57 ms	39.6
Swin ResUNet3+	6.2 min	71 ms	47.8
+ Quantum-Inspired Module	+0.4 min	+5 ms	+1.3
+ SpinalNet (Final Model)	+0.2 min	+3 ms	−2.1
**Proposed Hybrid Model (Total)**	**6.8 min**	**79 ms**	**47** **.** **0**

Bold values indicate the best performance across all models/metrics.

#### 5.8.2 Clinical deployment perspective

Clinically, even in high-throughput screening conditions, near-real-time analysis is made possible by inference latency of less than 100 ms per image. The overall processing time for a standard screening session with many mammography views stays within clinically acceptable bounds. Training is conducted offline and has no bearing on the viability of deployment.

Crucially, quantifiable improvements in sensitivity and robustness—especially for minor lesions, which are therapeutically more important than slight increases in inference time—justify the computational burden brought about by the quantum-inspired and SpinalNet components.

### 5.9 Statistical significance analysis of performance improvements

#### 5.9.1 Confidence intervals and hypothesis testing

We conducted paired statistical tests between the proposed framework and baseline and state-of-the-art techniques in order to extensively check the statistical significance of the reported performance gains. To ensure that paired samples matched identical test cases across models, all evaluations were performed at the case level. For each metric-accuracy, sensitivity, specificity, Dice coefficient, and IoU-95% CI was computed using non-parametric bootstrapping with 1,000 resamples. The reasoning behind such a choice is that it does not assume normalcy and works effectively for medical imaging datasets that are bound by small sample sizes. Finally, the proposed framework was compared to the transformer-based model (Swin ResUNet3+) and the strongest baseline (EfficientNet + UNet++) using paired t-tests. A criterion of *p* < 0.05 was used to determine statistical significance. Table 13 provides Statistical Analysis.

**Table 13:** Statistical analysis.

Metric	Proposed model (Mean ± SD)	95% CI	Baseline model	*p*-value
Accuracy (%)	93.8 ± 1.4	[91.1, 95.9]	92.3	0.013
Sensitivity (%)	94.1 ± 1.2	[92.0, 96.0]	90.1	0.004
Specificity (%)	92.7 ± 1.5	[90.2, 95.1]	92.0	0.041
Dice Coefficient	0.89 ± 0.02	[0.86, 0.92]	0.87	0.009
IoU (Jaccard)	0.82 ± 0.03	[0.78, 0.86]	0.79	0.011

#### 5.9.2 Clinical interpretation of statistical significance

In breast cancer screening, increased sensitivity is crucial as it reduces the number of missed malignancies. Additionally, consistent performance across test scenarios is indicated by the tight confidence intervals. More precise lesion border delineation is demonstrated by advancements in Dice and IoU, which is clinically significant for determining lesion magnitude and directing subsequent diagnostic choices.

### 5.10 Simulated real-world validation and robustness analysis

#### 5.10.1 Motivation and clinical context

Standardized benchmarks are provided by publicly accessible mammography datasets, however there are additional difficulties in real-world clinical settings, such as acquisition noise, class imbalance, varied imaging methods, and incorrect lesion presentation. We carried out a number of simulated real-world validation tests intended to stress-test the proposed framework under clinically relevant circumstances in order to close this gap.

#### 5.10.2 Noise and artifact robustness

Gaussian noise with increasing variance and contrast perturbations was applied to test images to simulate acquisition variability and scanner noise. To measure stability under inferior imaging configurations that are frequently encountered in clinical workflows, performance deterioration was evaluated.

#### 5.10.3 Simulating class imbalance

Malignant cases make up a tiny portion of exams in clinical screening populations, which are naturally unbalanced. We maintained a fixed training model while assessing model performance under more unbalanced test distributions to recreate this situation. Table 14 provides Robustness Analysis.

**Table 14:** Robustness analysis.

Scenario	Sensitivity (%)	Specificity (%)	Dice	IoU
Clean test data	94.1	92.7	0.89	0.82
+ Gaussian noise (*σ* = 0.05)	92.8	92.3	0.87	0.80
+ Gaussian noise (σ = 0.10)	90.9	91.6	0.85	0.78
Contrast reduction (−15%)	91.7	92.1	0.86	0.79
Class imbalance (1:8 malignant:benign)	92.4	93.2	0.88	0.81
Class imbalance (1:15 malignant:benign)	91.1	94.0	0.86	0.79

**Table 15:** Controlled comparison of quantum and classical classifiers.

Classifier (Same preprocessing & features)	Accuracy (%)	Sensitivity (%)	Specificity (%)	Dice	IoU
FCNN (Baseline)	91.6	88.7	92.4	0.85	0.77
MLP	92.0	89.5	92.6	0.86	0.78
Classical SpinalNet	92.7	91.2	92.8	0.87	0.80
**DQNN (Quantum-inspired)**	**93** **.** **4**	**93** **.** **6**	**92** **.** **9**	**0** **.** **88**	**0** **.** **81**

Bold values indicate the best performance across all models/metrics.

#### 5.10.4 Interpretation and clinical implications

The proposed model maintains excellent sensitivity even in highly imbalanced scenarios and exhibits graceful degradation as noise levels increase. Notably, despite actual data perturbations, sensitivity stays over 90% under all simulated settings, demonstrating resilience in identifying malignant instances. The results presented imply that the model can withstand typical clinical difficulties, such as skewed class distributions and noisy acquisitions.

#### 5.10.5 Limitations and future clinical validation

We recognize that prospective clinical validation cannot be entirely replaced by virtual studies. To evaluate usability, latency, and radiologist engagement, future work will test on multi-center hospital datasets and integrate into real-time clinical processes.

### 5.11 Controlled comparison of quantum and classical classifiers

We conducted a controlled comparison between the proposed Deep Quantum Neural Network (DQNN) and conventional neural classifiers utilizing the identical preprocessing pipeline, feature representations, and training strategy in order to objectively evaluate the contribution of the quantum-inspired decision mechanism. Only the classification module was changed, while all upgraded inputs (NLM + ET + ET-SegNet outputs and fused deep features from Swin ResUNet3+) were the same. In particular, the DQNN was contrasted with a multilayer perceptron (MLP), a traditional SpinalNet without quantum encoding, and a normal fully connected neural network (FCNN). Accuracy, sensitivity, specificity, Dice coefficient, and IoU were used to assess performance at the case level.

The results show that the DQNN consistently obtains greater sensitivity and segmentation overlap measures, indicating superior generalization to subtle and ambiguous lesion patterns, while traditional classifiers gain from the improved preprocessing workflow. This demonstrates that the quantum-inspired probabilistic feature modification, not only preprocessing, is responsible for the observed benefits. The detailed performance metrics and comparative analysis for the evaluated models are presented in Table 15.

#### 5.11.1 Interpretation and generalization perspective

In terms of sensitivity, which is crucial for reducing missed cancers in screening conditions, the DQNN clearly outperforms traditional classifiers. Further evidence that the quantum-inspired representation captures more discriminative lesion features comes from the enhanced Dice and IoU scores. The results obtained corroborate the theory that, even with robust preprocessing and feature extraction, quantum-inspired probabilistic encoding improves generalization beyond what is possible with traditional neural decision limits.

## 6 Conclusion

To automate breast cancer detection in mammograms, this study proposes a new model that utilizes deep quantum frameworks to address three significant limitations identified in previous research. These major issues were a lack of accuracy in segmenting images, a lack of understandability of images, and a lack of ability of earlier systems to generalize different results from various databases. The new framework, named Q-SpinalNet, incorporates SpinalNet and the frameworks of a Deep Quantum Neural Network, along with a multi-scale segmentation model named Swin ResUNet3+. The model provided solid results based on two major datasets, namely, DDSM and CBIS-DDSM, through a combination of end-to-end processing that consisted of a three-stage pre-processing system involving CEAMF-based denoising, Z-normalization, and relevant context enhancement. The system consisted of a combination of a segmentation model named Swin ResUNet3+, a classification model, and a combination of various processing techniques. The accuracy, sensitivity, specificity, precision, F-measure, Dice score, and IoU of this model were found to be 93.8%, 94.1%, 92.7%, 91.2%, 92.6%, 0.89, and 0.82, respectively.

The hybrid model put forward aims to balance the practical requirements of segmentation and classification improvements along with clinically interpretable decision-making for computer-aided diagnosis systems. Merging attention visualization, quantum probabilistic reasoning, and biologically inspired decision refining provides transparency and confidence in diagnostic results—a crucial component for medical adoption. In summary, Quantum-SpinalNet presents a diagnostic framework that is scalable, comprehensible, and computationally effective for mammographic breast cancer detection. The translational potential of this architecture will be further emphasized by future research concentrating on its extension to multi-modal imaging (ultrasound and MRI) and real-time clinical validation through a range of patient groups.

## Data Availability

Publicly available datasets were analyzed in this study. The datasets can be accessed at:
CBIS-DDSM: https://www.cancerimagingarchive.net/collection/cbis-ddsm/ DDSM: http://www.eng.usf.edu/cvprg/Mammography/Database.html
