# Correction: Quantum-SpinalNet: a hybrid deep learning approach for mammographic breast cancer detection

**DOI:** 10.3389/fdgth.2026.1892300

**Published:** 2026-08-05

**Authors:** Martina Jaincy D E, Venkatasubbu Pattabiraman

**Affiliations:** School of Computer Science and Engineering, Vellore Institute of Technology, Chennai, Tamil Nadu, India

**Keywords:** breast cancer detection, hybrid deep learning, mammography, quantum neural network, Swin ResUnet3+

Equation 2 in **4.2 Data pre-processing**, *4.2.3 Implementation consistency* was erroneously given as $U=\mathrm{mini}\left(2\left(e{?}^{\frac{a_{?}^{near(y)}}{sdis_{yt}^{near(y)}}}-1\right)+3,Sdis\right)$

The correct equation is $U=\min\left(2\left(e^{\frac{a_{\mu }^{near(y)}}{sdis_{yt}^{near(y)}}}-1\right)+3,Sdis\right)$

Equation 4 in **4.2 Data pre-processing**, *4.2.3 Implementation consistency* was erroneously given as

$$U=mini\left(2\left[log_{10\left(\frac{sdis_{\;ji}^{near(y)}}{a_{\;ji}^{near(y)}}\right)}\right]+3,Sdis\right)$$

The correct equation is $U=min\left(2\left[log_{10\left(\frac{sdis_{\;ji}^{near(y)}}{a_{\;ji}^{near(y)}}\right)}\right]+3,Sdis\right)$

The equation in **4.2 Data pre-processing**, *4.2.3 Implementation,* Algorithm 1, line 8 was erroneously given as$U=\mathrm{mini}\left(2\left(e{?}^{\frac{a_{?}^{near(y)}}{sdis_{yt}^{near(y)}}}-1\right)+3,Sdis\right)$

The correct equation is $U=\min\left(2\left(e^{\frac{a_{\mu }^{near(y)}}{sdis_{yt}^{near(y)}}}-1\right)+3,Sdis\right)$

The equation **4.2 Data pre-processing**, 4.2.3 Implementation, Algorithm 1, line 10 was erroneously given as $U=mini\left(2\left[log_{10\left(\frac{sdis_{\;ji}^{near(y)}}{a_{\;ji}^{near(y)}}\right)}\right]+3,Sdis\right)$

The correct equation is $U=min\left(2\left[log_{10\left(\frac{sdis_{\;ji}^{near(y)}}{a_{\;ji}^{near(y)}}\right)}\right]+3,Sdis\right)$

“These networks can be utilized for image classification and segmentation, as they can process data efficiently and quickly identify abnormalities” has been changed to “These networks could be utilized in image classification and segmentation as can process statistics and quickly identify any issues”.

A correction has been made to the section 1 **Introduction**, Paragraph Number 5.

“Image” has been changed to “photograph”.

A correction has been made to the section 1 **Introduction**, Paragraph Number 6.

“photo classification” has been changed to “image classification”.

A correction has been made to the section 1 Introduction,Paragraph number 5 and 6.

“The advance of technology has seen the use of models such as picture processing, system gaining knowledge of (ML), and deep learning (DL) in medical imaging” should be “The advance of technology has seen the use of models such as image processing, machine learning (ML), and deep learning (DL) in medical imaging”.

A correction has been made to the section **1 Introduction**, Paragraph Number 4.

“manual characteristic extraction to computerized feature identity” has been changed to “manual feature extraction to automated feature learning”

A correction has been made to the section **1 Introduction**, Paragraph Number 5.

“medical picture” has been changed to “medical image”.

A correction has been made to the section **1 Introduction**, *1.4 Structural innovation: SpinalNet integration*, Paragraph Number 5.

“U-internet” has been changed to “U-Net”.

A correction has been made to the section **2 Literature survey**, Paragraph Number 4.

“mammogram photos” has been changed to “mammogram images”.

A correction has been made to the section **2 Literature survey**, Paragraph Number 4.

“Picture artifacts” has been changed to “image artifacts”.

A correction has been made to the section **2 Literature survey**, Paragraph Number 4.

“mammographic photos has been changed to “mammographic images”.

A correction has been made to the section **2 Literature survey**, Paragraph Number 6.

“education and evaluation sets” has been changed to “training and testing sets”.

A correction has been made to the section **2 Literature survey**, Paragraph Number 7.

“deep mastering techniques” has been changed to “deep learning techniques”.

A correction has been made to the section **2 Literature survey**, Paragraph Number 8.

“photographs” has been changed to “images”.

A correction has been made to the section **2 Literature survey**, Paragraph Number 8.

“Constraints in picture quality, segmentation accuracy, and powerful function extraction are the main issues” has been changed to “Constraints in image quality, segmentation accuracy, and feature extraction are the main issues”.

A correction has been made to the section **2 Literature survey**, Paragraph Number 9.

“mammography photos” has been changed to “mammographic images”.

A correction has been made to the section **2 Literature survey**, Paragraph Number 9.

“picture normalization” has been changed to “image normalization”.

A correction has been made to the section **3 System architecture**, Paragraph Number 2.

“mastering strategies” has been changed to “learning techniques”.

A correction has been made to the section **3 System architecture**, Paragraph Number 2.

“pictures” has been changed to “images”.

A correction has been made to the section **4 proposed system**,

*4.1 Data acquisition*, Paragraph Number 1.

“picture filtering” has been changed to “image filtering”.

A correction has been made to the section **4 proposed system**, *4.1 Data acquisition*, Paragraph Number 1.

“characteristic extraction” has been changed to “feature extraction”.

A correction has been made to the section **4 proposed system**,

*4.1 Data acquisition*, Paragraph Number 1.

“NNN pixels” has been changed to “N pixels”.

A correction has been made to the section **4 Proposed system**, *4.1.2 Image normalization*, Paragraph Number 1.

“Here, γy is the standard deviation” has been changed to “Here, Δy is the standard deviation”

A correction has been made to the section **4 Proposed system**, *4.1.2 Image normalization*, Paragraph Number 1.

“ene_mean_” has been changed to “enemean”.

A correction has been made to the section **4 Proposed system**, *4.1.3 Context-based image contrast improvement*, Paragraph Number 3.

“LO_low_” has been changed to “(\(LO_ {low}\))”.

A correction has been made to the section **4 Proposed system**, *4.1.3 Context-based image contrast improvement*, Paragraph Number 3.

“A_b_” has been changed to “Ab”.

A correction has been made to the section **4 Proposed system**, *4.4.2 Q-SpinalNet layer,* Paragraph Number 1.

“ωₙₜ is the weight and ξₜ is the input from the i_th _neuron.” has been changed to “*ω*mi is the weight, and *ξ*i is the input from the ith neuron”.

A correction has been made to the section ***4 Proposed system**, 4.4.3 Spinalnet model*, Paragraph Number 3.

“picture resolution” has been changed to “ image resolution”.

A correction has been made to the section 4.3 Tumor segmentation using Swin ResUnet3+ Paragraph Number 1

“picture Z” has been changed to “ image Z”.

A correction has been made to the section 4.3 Tumor segmentation using Swin ResUnet3+ Paragraph Number 2

“mammography (MG) picture2 has been changed to “mammographic image”.

A correction has been made to the section **4 Proposed system**, *4.4 BC detection using Q-SpinalNet*, Paragraph Number 2.

“buried layer” has been changed to “hidden layer”.

A correction has been made to the section **4 Proposed system**, *4.4.3 Spinalnet model*, Paragraph Number 4.

“medical picture categorization” has been changed to “medical image classification”.

A correction has been made to the section **4 Proposed system**, *4.6 Synergy between quantum-inspired modeling and SpinalNet*, Paragraph Number 1.

“4.4.4 Overall method for BC identification using MG pictures” has been changed to “4.4.4 Overall method for BC identification using MG images”.

A correction has been made to the section 4.4 BC detection using Q-SpinalNet, 4.4.4 Overall method for BC identification using MG pictures.

“picture quality” has been changed to “image quality”.

A correction has been made to the section 4.4.5 End-to-end processing chain: explicit algorithmic separation, 4.4.5.1 Chain-1: classical image conditioning and feature augmentation (pre-segmentation stage), Paragraph Number 1.

“picture” has been changed to “image”.

A correction has been made to the section 4.4.5 End-to-end processing chain: explicit algorithmic separation, 4.4.5.2 Chain-2: final decision stage, Paragraph Number 1.

“Swin Transformer's Attention Maps, which can be visualized for radiologists to study, reveal which picture regions affected judgments.” has been changed to “The Swin Transformer's attention maps, which can be visualized for radiologists, reveal the image regions that most strongly influenced the model's decisions.”

A correction has been made to the section **5 Performance metrics**, *5.2 Statistical analysis performed on the proposed Q-SpinalNet*, Paragraph Number 1.

“mammography pictures” has been changed to “mammographic images”.

A correction has been made to the section **5 Performance metrics**, *5.2 Statistical analysis performed on the proposed Q-SpinalNet*, Paragraph Number 2.

“judgments of the model” has been changed to “model decisions”.

A correction has been made to the section **5 Performance metrics**, *5.4.3 Attention map sanity checks*, Paragraph Number 1.

“input pictures” has been changed to “input images”.

A correction has been made to the section **5 Performance metrics**, *5.6.1 Ground truth source for segmentatio*n, Paragraph Number 1.

“where P represents the predicted segmentation mask and G represents the ground truth mask.” has been changed to “where \(P\) represents the predicted segmentation mask and \(G\) represents the ground truth mask.”

A correction has been made to the section **5 Performance metrics,** *5.6.2 Dice coefficient and IoU (Jaccard index) calculation*, Paragraph Number 1.

“photos” has been changed to “input images”.

A correction has been made to the section **5 Performance metrics**, *5.6.3 Per-Image and per-case assessment procedure*, Paragraph Number 1.

“picture” has been changed to “image”.

A correction has been made to the section 5.7 Patient-level data splitting and leakage prevention, Paragraph Number 2.

“Strict patient/case-level data separation was used in all trials to guarantee objective assessment and avoid knowledge leaking” has been changed to “Strict patient-/case-level data separation was used in all trials to ensure objective assessment and prevent data leakage”.

A correction has been made to the section 5.7 Patient-level data splitting and leakage prevention, Paragraph Number 1.

“photos” has been changed to “images”.

Correction have been made to the section **5 Performance metrics**, *5.7 Patient-level data splitting and leakage prevention, 5.7.1 Prevention of patch-level and view-level leakage, 5.7.2 Cross-validation protocol* and **5 Performance metrics***, 5.7.3 Clinical justification*.

“Photos” has been changed to “images”.

A correction has been made to the section **5 Performance metrics,** *5.7.3 Clinical justification*, Paragraph Number 1.

“picture” has been changed to “image”.

A correction has been made to the section 5.8 Computational complexity and clinical feasibility, 5.8.2 Clinical deployment perspective Paragraph Number 1.

“test pictures” has been changed to “test images”.

A correction has been made to the section **5 Performance metrics**, *5.10.2 Noise and artifact robustness*, Paragraph Number 1.

The original version of this article has been updated.

